# Supplementary material for: Paying attention to cardiac surgical risk: An interpretable machine learning approach using an uncertainty-aware attentive neural network
Source: PLoS One. 2023 Aug 30;18(8):e0289930. doi: 10.1371/journal.pone.0289930 (PMC10468047; doi:10.1371/journal.pone.0289930)
Supplement: S1 Table — A description of how each variable was defined. (DOCX) [file pone.0289930.s001.docx]

**S1 Table: Variable Definitions**

| Variable | Definition in ANZSCTS Database | Definition in MIMIC III Database |
| --- | --- | --- |
| Age | Time from recorded date of birth in years (range 0 - 110) | Time from recorded date of birth in years |
| Sex | Recorded biological sex | Recorded biological sex |
| Body Mass Index | Recorded weight (kg) / height (m) squared (range 5 - 90) | Recorded weight (kg) / height (m) squared |
| Indigenous Status | Whether the patient was identified as indigenous australia | NA |
| Insurance | The insurance status of the patient at time of surgery | The insurance status of the patient at time of hospital admission |
| Previous Cardiac Procedure | A recorded history of previous cardiothoracic intervention (surgical or percutaneous) | NA |
| History of Arrhythmia | A clinically documented pre-operative arrhythmia (including AF, heart block, VT or VF) | Any recorded ICD-9 code with suffix 428* |
| History of Smoking | Confirmed history of tobacco use | Any recorded ICD-9 code with suffix V15* |
| Current Smoker | Use of tobacco within one month of surgery | NA |
| History of Diabetes | Any recorded history of diabetes | ICD-9 codes for Type 1 including '25001','25003','25011','25013','25021','25023','25031','25033','25041','25043','25051','25053','25061','25063','25071','25073','25081','25083','25091','25093',  and Type 2 including '25000','25002','25010','25012','25020','25022','25030','25032','25040','25042','25050','25052','25060','25062','25070','25072','25080','25082','25090','25092' |
| Diabetes Control | Whether the patient was diet, oral medication, or insulin controlled. | NA |
| History of Hypercholesterolaemia | Recorded history of high cholesterol | ICD-9 code witCh suffix 272 |
| History of Hypertension | Recorded history of high blood pressure | ICD-9 code 401-405 |
| Lung Disease Severity | Recorded history of lung disease and whether patient requires oral bronchodilator therapy, chronic oral steroids for lung disease, or room air pO2 < 60 or room air pCO2 >50 | NA |
| Type of Cerebrovascular Disease | Recorded history of cerebrovascular disease and the type, including coma, stroke or TIA, and carotid stenosis | NA |
| History of Peripheral Vascular Disease | Recorded history of peripheral vascular disease | ICD-9 codes for PVD including suffixes 440* and 441*, and codes '0930','4373','4471','5571','5579','V434', ‘4431 - 4439’ |
| History of Heart Failure | Recorded history of heart failure | ICD-9 codes suffiexed with 428* and codes '39891','40201','40211','40291','40401','40403','40411','40413','40491','40493' or between ‘4254’ - ‘4259’ |
| History of Dialysis | A preoperative requirement for renal replacement therapy | NA |
| Previous Renal Transplant | A recorded history or renal transplant | NA |
| Procedure Number | The procedure number of the operation if the patient had undergone previous procedures this admission | NA |
| Infective Endocarditis | Whether the patient had presented with valvular disease of infectious aetiology | NA |
| Admitted with Heart Failure | Whether the patient was admitted to hospital in heart failure | NA |
| Ejection Fraction (%) | Preoperative left ventricular ejection fraction (from LV gram, echo, radionucleotide or MRI imaging) (range 0 - 100) | NA |
| EF Estimate | If a specific number was not reported, an estimate was input which was one of LVEF >60%, 46-60%, 30-40%, or <30% | NA |
| Preoperative Shock | Whether the patient was in cardiogenic shock prior to and at the time of the procedure | NA |
| NYHA Class | The NHYA Class (I-IV) at the time of the operation | NA |
| Operative Urgency | Whether the operation was elective, urgent, emergent, or salvage | NA |
| Type of Operation | One of CABG alone, Valve alone, CABG+Valve, or Other | NA |
| Aortic Valve | The operation involved the aortic valve | NA |
| Mitral Valve | The operation involved the mitral valve | NA |
| Tricuspid Valve | The operation involved the tricuspid valve | NA |
| Pulmonary Valve | The operation involved the pulmonary valve | NA |
| Cross Clamp Time (mins) | The total time in minutes that the aorta was clamped during bypass  (range 0 - 600) | NA |
| Bypass Time (mins) | The total time for cardiopulmonary bypass (range 1 - 999) | NA |
| Preoperative Creatinine (micromol/L) | The latest preoperative creatinine prior to the operation (range 20-2000) | Latest recorded creatinine in the dataset prior to ICU admission |
| Estimated Filtration Rate (mL/min per 1.73m2) | Derived variable. Calculated from the PRECR, Age, and Sex using the following modified MDRD calculation:  175 x [PRECR x 0.0113]^-1.154 x Age(years)^-0.203 (x 0.742 if female) | NA |
| Preoperative Haemaglobin (g/L) | The latest haemaglobin prior to the operation (range 40-200) | NA |
| Lowest Intraoperative Haemaglobin (g/L) | The lowest haemaglobin recorded during the operation (range 40 - 200) | NA |
| Hours in ICU | Number of hours the patient was in ICU, as a difference of discharge time - admit time (range 0 - 1000) | Number of hours the patient was in ICU, as a difference of discharge time - admit time |
| Hours Ventilated | Number of hours ventilated measures as extubation time - admit time  (range 0 - 1000) | NA |
| Drain output in first 4 hours (mL) | Recorded drain output in the first four hours after the operation (range 0 - 4000) | NA |
