# Supplementary material for: Paying attention to cardiac surgical risk: An interpretable machine learning approach using an uncertainty-aware attentive neural network
Source: PLoS One. 2023 Aug 30;18(8):e0289930. doi: 10.1371/journal.pone.0289930 (PMC10468047; doi:10.1371/journal.pone.0289930)
Supplement: S2 Table — A description of how each outcome was defined. (DOCX) [file pone.0289930.s002.docx]

**S2 Table: Outcome Definitions**

| Outcome | Definition in ANZSCTS Database | Definition in MIMIC III Database |
| --- | --- | --- |
| 30 Day Mortality (MORT30) | Death within 30 days of the procedure | Death within 30 days of admission to ICU |
| Readmission to ICU (REICU) | Whether the patient was readmitted to ICU during that hospital admission |  |
| Reintubation (REINT) | Whether the patient was reintubated after extubation during the index admission |  |
| Postoperative Acute Kidney Injury (NRF) | 1. Increased serum creatinine to >0.2mmol/L AND a doubling or greater increase in creatinine over the baseline preoperative value AND the patient did no require renal replacement therapy preoperatively; OR  2. A new requirement for dialysis postoperatively |  |
| New dialysis requirement (HAEMOFIL) | New postoperative renal replacement requirement |  |
| New postoperative arrhythmia (NARRT) | A new form of cardiac arrhythmia occuring postoperatively and required treatment |  |
| Deep sternal wound infection (INFDS) | Infection of sternal bone, muscle and/or mediastinum requiring debridement and including one of positive blood cultures OR treatment with antibiotics |  |
| Return to theatre (RTT) | Did the patient require a return to the operating theatre during the admission, including any procedures in ICU that would normally be performed in the operating room |  |
