# Supplementary material for: Paying attention to cardiac surgical risk: An interpretable machine learning approach using an uncertainty-aware attentive neural network
Source: PLoS One. 2023 Aug 30;18(8):e0289930. doi: 10.1371/journal.pone.0289930 (PMC10468047; doi:10.1371/journal.pone.0289930)
Supplement: S3 Table — A complete table of patient characteristics for all variables included in the analysis. (DOCX) [file pone.0289930.s003.docx]

**S3 Table: Patient Characteristics of the Database Subsets**

| **Variable** | **ANZSCTS Subset**  **Mean (SD) or %** | **MIMIC III Subset**  **Mean (SD) or %** | **Missingness %**  **(ANZSCTS / MIMIC)** |
| --- | --- | --- | --- |
| Age (years) | 65.61 (12.89) | 69.57 (27.69) | 0.02/1.05 |
| Sex | 73.20% | 68.19% | 0.00/0.00 |
| Body Mass Index | 28.48 (5.43) | 28.59 (5.77) | 0.30/7.22 |
| Indigenous Status | 2.62% |  | 1.19 |
| Insurance |  |  | 1.31/0.00 |
| Private | 25.47% | 36.63% |  |
| DVA | 1.34% | 0% |  |
| Medicare | 71.26% | 56.50% |  |
| Self Insured | 0.30% | 0.39% |  |
| Overseas | 0.52% | 0% |  |
| Other | 0.44% | 6.47% |  |
| Previous Cardiac Procedure | 19.75% |  | 0.15 |
| History of Arrhythmia | 17.17% | 52.34% | 0.18/0.30 |
| History of Smoking | 57.35% | 15.92% | 0.16/0.00 |
| Current Smoker | 23.22% |  | 41.75 |
| History of Diabetes | 29.19% | 33.26% | 0.17/0.00 |
| Diabetes | 3.06% |  | 70.57 |
| History of Hypercholesterolaemia | 66.08% | 55.07% | 0.19/0.00 |
| History of Hypertension | 72.08% | 70.26% | 0.18/0.00 |
| Lung Disease Severity |  |  | 0.00 |
| Mild (chronic puffer use) | 69.39% |  |  |
| Moderate (chronic oral steroid) | 22.66% |  |  |
| Severe Room air p0 2 < 60 or room air pC0 2 > 50 | 7.94% |  |  |
| Type of Cerebrovascular Disease |  |  | 0.00 |
| Coma | 0.40% |  |  |
| CVA | 49.83% |  |  |
| RIND/TIA | 33.36% |  |  |
| Carotid Occlusive Disease | 16.39% |  |  |
| History of Peripheral Vascular Disease | 8.93% | 14.88% | 0.18/0.30 |
| History of Heart Failure | 20.99% | 29.93% | 0.17/0.30 |
| History of Dialysis | 1.65% |  | 0.15 |
| Previous Renal Transplant | 0.50% |  | 17.68 |
| Procedure Number | 1.04 (0.23) |  | 0.00 |
| Infective Endocarditis |  |  | 0.00 |
| Active | 66.88% |  |  |
| Treated | 33.12% |  |  |
| Admitted with Heart Failure | 43.26% |  | 77.39 |
| Ejection Fraction (%) | 54.69 (14.12) |  | 50.86 |
| EF Estimate |  |  | 2.51 |
| >60 | 51.48% |  |  |
| 46-60 | 30.74% |  |  |
| 30-45 | 13.17% |  |  |
| <30 | 4.62% |  |  |
| Preoperative Shock | 2.10% |  | 0.16 |
| NYHA Class |  |  | 2.62 |
| Class 1 | 37.58% |  |  |
| Class 2 | 35.47% |  |  |
| Class 3 | 21.26% |  |  |
| Class 4 | 5.69% |  |  |
| Operative Urgency |  |  | 0.06 |
| Elective | 68.72% |  |  |
| Urgent | 26.52% |  |  |
| Emergent | 4.41% |  |  |
| Salvage | 0.35% |  |  |
| Type of Operation |  |  | 0.15/0.00 |
| CABG alone | 53.46% | 58.18% |  |
| Isolated Valve | 20.47% | 26.27% |  |
| CABG+Valve | 10.20% | 15.55% |  |
| Other | 15.87% | 0% |  |
| Aortic Valve | 26.91% |  | 0.00 |
| Mitral Valve | 13.39% |  | 0.00 |
| Tricuspid Valve | 3.07% |  | 0.00 |
| Pulmonary Valve | 0.75% |  | 0.00 |
| Cross Clamp Time (mins) | 78.00 (43.75) |  | 5.23 |
| Bypass Time (mins) | 110.42 (58.87) |  | 5.21 |
| Preoperative Creatinine (micromol/L) | 101.77 (85.71) | 109.65 (126.57) | 0.00/0.00 |
| Estimated Filtration Rate (mL/min per 1.73m2) | 68.51 (21.26) |  | 0.00 |
| Preoperative Haemaglobin (g/L) | 135.09 (19.08) |  | 68.46 |
| Lowest Intraoperative Haemaglobin (g/L) | 92.08 (19.88) |  | 72.57 |
| Hours in ICU | 83.80 (147.93) | 92.67 (129.66) | 0.00/0.00 |
| Hours Ventilated | 47.04 (162.45) |  | 0.00 |
| Drain output in first 4 hours (mL) | 23.69 (71.15) |  | 2.43 |
