# Supplementary material for: Paying attention to cardiac surgical risk: An interpretable machine learning approach using an uncertainty-aware attentive neural network
Source: PLoS One. 2023 Aug 30;18(8):e0289930. doi: 10.1371/journal.pone.0289930 (PMC10468047; doi:10.1371/journal.pone.0289930)
Supplement: S5 Table — A complete table for all internal validation and external validation results for uncertainty calibration. (DOCX) [file pone.0289930.s005.docx]

**S5 Table: Uncertainty Calibration Across All Datasets and Outcomes**

| Outcome | Model | Dataset | AUC High Confidence | AUC Aleatoric | AUC Epistemic | Brier Score |
| --- | --- | --- | --- | --- | --- | --- |
| REICU | UAN-GVI | train | 0.72 (0.03) | 1.00 (0.00) | 0.58 (0.01) | 0.23 (0.00) |
| REICU | UAN-GVI | test | 0.71 (0.06) | 1.00 (0.00) | 0.61 (0.11) | 0.22 (0.01) |
| REICU | UAN-GVI | val | 0.70 (0.06) | 1.00 (0.00) | 0.61 (0.11) | 0.22 (0.01) |
| REICU | UAN-GVI | val_p | 0.65 (0.06) | 1.00 (0.00) | 0.61 (0.11) | 0.23 (0.01) |
| REICU | UAN-PN | train | 0.58 (0.16) | 1.00 (0.00) | 0.58 (0.01) | 0.23 (0.00) |
| REICU | UAN-PN | test | 0.54 (0.09) | 1.00 (0.00) | 0.62 (0.10) | 0.23 (0.02) |
| REICU | UAN-PN | val | 0.52 (0.08) | 1.00 (0.00) | 0.62 (0.10) | 0.22 (0.02) |
| REICU | UAN-PN | val_p | 0.51 (0.13) | 1.00 (0.00) | 0.61 (0.11) | 0.23 (0.02) |
| REICU | LR | train | 0.69 (0.00) | 0.99 (0.01) | 0.48 (0.00) | 0.23 (0.00) |
| REICU | LR | test | 0.68 (0.01) | 1.00 (0.00) | 0.70 (0.01) | 0.23 (0.00) |
| REICU | LR | val_p | 0.68 (0.00) | 1.00 (0.00) | 0.69 (0.01) | 0.23 (0.00) |
| REICU | LR-SI | train | 0.71 (0.00) | 1.00 (0.00) | 0.48 (0.00) | 0.22 (0.00) |
| REICU | LR-SI | test | 0.70 (0.01) | 1.00 (0.00) | 0.72 (0.02) | 0.22 (0.00) |
| REICU | LR-SI | val | 0.69 (0.00) | 1.00 (0.00) | 0.72 (0.01) | 0.22 (0.00) |
| REICU | LR-MICE | train | 0.71 (0.00) | 1.00 (0.01) | 0.48 (0.00) | 0.22 (0.00) |
| REICU | LR-MICE | test | 0.71 (0.01) | 1.00 (0.00) | 0.72 (0.01) | 0.22 (0.00) |
| REICU | LR-MICE | val | 0.68 (0.00) | 1.00 (0.00) | 0.71 (0.01) | 0.22 (0.00) |
| REICU | XGBoost | train | 0.79 (0.03) | 0.95 (0.07) | 0.57 (0.04) | 0.20 (0.01) |
| REICU | XGBoost | test | 0.71 (0.02) | 0.99 (0.01) | 0.74 (0.07) | 0.17 (0.05) |
| REICU | XGBoost | val | 0.70 (0.01) | 0.99 (0.01) | 0.74 (0.07) | 0.18 (0.05) |
| REICU | XGBoost-SI | train | 0.83 (0.01) | 0.95 (0.06) | 0.60 (0.04) | 0.19 (0.01) |
| REICU | XGBoost-SI | test | 0.73 (0.01) | 0.99 (0.01) | 0.75 (0.07) | 0.17 (0.05) |
| REICU | XGBoost-SI | val | 0.72 (0.01) | 0.99 (0.01) | 0.75 (0.07) | 0.17 (0.05) |
| REICU | XGBoost-MICE | train | 0.83 (0.01) | 0.95 (0.07) | 0.60 (0.04) | 0.19 (0.01) |
| REICU | XGBoost-MICE | test | 0.73 (0.01) | 0.99 (0.01) | 0.74 (0.08) | 0.17 (0.05) |
| REICU | XGBoost-MICE | val | 0.72 (0.01) | 0.98 (0.01) | 0.75 (0.07) | 0.17 (0.05) |
| REINT | UAN-GVI | train | 0.84 (0.01) | 1.00 (0.00) | 0.63 (0.03) | 0.19 (0.00) |
| REINT | UAN-GVI | test | 0.88 (0.01) | 1.00 (0.00) | 0.72 (0.07) | 0.16 (0.02) |
| REINT | UAN-GVI | val | 0.90 (0.01) | 1.00 (0.00) | 0.73 (0.08) | 0.16 (0.02) |
| REINT | UAN-GVI | val_p | 0.89 (0.01) | 1.00 (0.00) | 0.78 (0.09) | 0.16 (0.02) |
| REINT | UAN-PN | train | 0.87 (0.07) | 1.00 (0.00) | 0.59 (0.03) | 0.19 (0.00) |
| REINT | UAN-PN | test | 0.89 (0.06) | 1.00 (0.00) | 0.66 (0.14) | 0.16 (0.01) |
| REINT | UAN-PN | val | 0.88 (0.10) | 1.00 (0.00) | 0.67 (0.14) | 0.16 (0.01) |
| REINT | UAN-PN | val_p | 0.85 (0.11) | 1.00 (0.00) | 0.64 (0.16) | 0.16 (0.02) |
| REINT | LR | train | 0.85 (0.00) | 0.99 (0.00) | 0.44 (0.00) | 0.17 (0.00) |
| REINT | LR | test | 0.85 (0.01) | 1.00 (0.00) | 0.71 (0.01) | 0.15 (0.00) |
| REINT | LR | val_p | 0.87 (0.00) | 1.00 (0.00) | 0.71 (0.01) | 0.16 (0.00) |
| REINT | LR-SI | train | 0.86 (0.00) | 0.99 (0.00) | 0.48 (0.00) | 0.17 (0.00) |
| REINT | LR-SI | test | 0.86 (0.00) | 1.00 (0.00) | 0.73 (0.01) | 0.15 (0.00) |
| REINT | LR-SI | val | 0.88 (0.00) | 1.00 (0.00) | 0.75 (0.01) | 0.15 (0.00) |
| REINT | LR-MICE | train | 0.86 (0.00) | 0.99 (0.00) | 0.49 (0.00) | 0.17 (0.00) |
| REINT | LR-MICE | test | 0.86 (0.00) | 1.00 (0.00) | 0.73 (0.01) | 0.15 (0.00) |
| REINT | LR-MICE | val | 0.88 (0.00) | 1.00 (0.00) | 0.74 (0.02) | 0.15 (0.00) |
| REINT | XGBoost | train | 0.91 (0.02) | 0.99 (0.01) | 0.74 (0.02) | 0.14 (0.01) |
| REINT | XGBoost | test | 0.86 (0.01) | 1.00 (0.00) | 0.71 (0.05) | 0.12 (0.02) |
| REINT | XGBoost | val | 0.89 (0.01) | 1.00 (0.00) | 0.70 (0.07) | 0.12 (0.02) |
| REINT | XGBoost-SI | train | 0.93 (0.00) | 0.99 (0.02) | 0.77 (0.01) | 0.13 (0.00) |
| REINT | XGBoost-SI | test | 0.88 (0.01) | 1.00 (0.00) | 0.77 (0.05) | 0.11 (0.02) |
| REINT | XGBoost-SI | val | 0.90 (0.00) | 1.00 (0.00) | 0.80 (0.05) | 0.10 (0.02) |
| REINT | XGBoost-MICE | train | 0.93 (0.00) | 0.98 (0.01) | 0.78 (0.01) | 0.13 (0.00) |
| REINT | XGBoost-MICE | test | 0.88 (0.01) | 1.00 (0.00) | 0.77 (0.07) | 0.11 (0.02) |
| REINT | XGBoost-MICE | val | 0.90 (0.00) | 1.00 (0.00) | 0.76 (0.07) | 0.11 (0.02) |
| RTT | UAN-GVI | train | 0.81 (0.01) | 1.00 (0.00) | 0.63 (0.01) | 0.21 (0.00) |
| RTT | UAN-GVI | test | 0.84 (0.01) | 1.00 (0.00) | 0.69 (0.07) | 0.19 (0.02) |
| RTT | UAN-GVI | val | 0.85 (0.01) | 1.00 (0.00) | 0.70 (0.06) | 0.18 (0.01) |
| RTT | UAN-GVI | val_p | 0.80 (0.02) | 1.00 (0.00) | 0.67 (0.10) | 0.20 (0.02) |
| RTT | UAN-PN | train | 0.78 (0.09) | 1.00 (0.00) | 0.59 (0.02) | 0.21 (0.00) |
| RTT | UAN-PN | test | 0.75 (0.11) | 1.00 (0.00) | 0.66 (0.10) | 0.19 (0.01) |
| RTT | UAN-PN | val | 0.75 (0.11) | 1.00 (0.00) | 0.66 (0.10) | 0.19 (0.01) |
| RTT | UAN-PN | val_p | 0.75 (0.12) | 1.00 (0.00) | 0.65 (0.10) | 0.20 (0.02) |
| RTT | LR | train | 0.77 (0.00) | 1.00 (0.00) | 0.46 (0.00) | 0.21 (0.00) |
| RTT | LR | test | 0.77 (0.01) | 1.00 (0.00) | 0.68 (0.01) | 0.20 (0.00) |
| RTT | LR | val_p | 0.77 (0.00) | 0.99 (0.01) | 0.68 (0.01) | 0.22 (0.00) |
| RTT | LR-SI | train | 0.79 (0.00) | 0.99 (0.01) | 0.46 (0.00) | 0.20 (0.00) |
| RTT | LR-SI | test | 0.79 (0.01) | 1.00 (0.00) | 0.71 (0.01) | 0.19 (0.00) |
| RTT | LR-SI | val | 0.79 (0.00) | 0.99 (0.00) | 0.69 (0.01) | 0.19 (0.00) |
| RTT | LR-MICE | train | 0.77 (0.00) | 1.00 (0.00) | 0.46 (0.00) | 0.20 (0.00) |
| RTT | LR-MICE | test | 0.77 (0.01) | 1.00 (0.00) | 0.71 (0.01) | 0.20 (0.00) |
| RTT | LR-MICE | val | 0.78 (0.00) | 0.99 (0.00) | 0.70 (0.01) | 0.20 (0.00) |
| RTT | XGBoost | train | 0.82 (0.02) | 0.97 (0.04) | 0.64 (0.02) | 0.18 (0.01) |
| RTT | XGBoost | test | 0.80 (0.02) | 0.99 (0.01) | 0.69 (0.06) | 0.16 (0.03) |
| RTT | XGBoost | val | 0.81 (0.02) | 0.99 (0.01) | 0.67 (0.07) | 0.17 (0.03) |
| RTT | XGBoost-SI | train | 0.86 (0.00) | 0.98 (0.03) | 0.67 (0.02) | 0.17 (0.01) |
| RTT | XGBoost-SI | test | 0.82 (0.00) | 0.99 (0.01) | 0.71 (0.07) | 0.15 (0.03) |
| RTT | XGBoost-SI | val | 0.84 (0.00) | 0.99 (0.01) | 0.67 (0.08) | 0.16 (0.03) |
| RTT | XGBoost-MICE | train | 0.85 (0.00) | 0.98 (0.03) | 0.68 (0.02) | 0.17 (0.01) |
| RTT | XGBoost-MICE | test | 0.82 (0.00) | 0.99 (0.01) | 0.71 (0.07) | 0.15 (0.03) |
| RTT | XGBoost-MICE | val | 0.83 (0.00) | 0.99 (0.01) | 0.70 (0.07) | 0.15 (0.03) |
| NRF | UAN-GVI | train | 0.84 (0.01) | 1.00 (0.00) | 0.65 (0.02) | 0.19 (0.00) |
| NRF | UAN-GVI | test | 0.87 (0.01) | 1.00 (0.00) | 0.71 (0.07) | 0.17 (0.01) |
| NRF | UAN-GVI | val | 0.88 (0.01) | 1.00 (0.00) | 0.72 (0.07) | 0.16 (0.01) |
| NRF | UAN-GVI | val_p | 0.86 (0.01) | 1.00 (0.00) | 0.69 (0.12) | 0.18 (0.02) |
| NRF | UAN-PN | train | 0.86 (0.07) | 1.00 (0.00) | 0.59 (0.02) | 0.19 (0.00) |
| NRF | UAN-PN | test | 0.85 (0.11) | 1.00 (0.00) | 0.67 (0.10) | 0.17 (0.01) |
| NRF | UAN-PN | val | 0.89 (0.07) | 1.00 (0.00) | 0.69 (0.10) | 0.17 (0.01) |
| NRF | UAN-PN | val_p | 0.83 (0.12) | 1.00 (0.00) | 0.68 (0.12) | 0.18 (0.02) |
| NRF | LR | train | 0.82 (0.00) | 0.99 (0.00) | 0.46 (0.00) | 0.19 (0.00) |
| NRF | LR | test | 0.82 (0.01) | 1.00 (0.00) | 0.67 (0.01) | 0.18 (0.00) |
| NRF | LR | val_p | 0.83 (0.00) | 1.00 (0.00) | 0.68 (0.01) | 0.19 (0.00) |
| NRF | LR-SI | train | 0.85 (0.00) | 0.99 (0.00) | 0.47 (0.00) | 0.17 (0.00) |
| NRF | LR-SI | test | 0.85 (0.01) | 1.00 (0.00) | 0.68 (0.02) | 0.16 (0.00) |
| NRF | LR-SI | val | 0.87 (0.00) | 1.00 (0.00) | 0.73 (0.01) | 0.15 (0.00) |
| NRF | LR-MICE | train | 0.84 (0.00) | 1.00 (0.00) | 0.48 (0.00) | 0.17 (0.00) |
| NRF | LR-MICE | test | 0.84 (0.01) | 1.00 (0.00) | 0.68 (0.01) | 0.17 (0.00) |
| NRF | LR-MICE | val | 0.85 (0.00) | 1.00 (0.00) | 0.73 (0.01) | 0.16 (0.00) |
| NRF | XGBoost | train | 0.89 (0.02) | 0.98 (0.03) | 0.71 (0.02) | 0.15 (0.01) |
| NRF | XGBoost | test | 0.86 (0.02) | 1.00 (0.00) | 0.72 (0.09) | 0.13 (0.03) |
| NRF | XGBoost | val | 0.88 (0.02) | 1.00 (0.00) | 0.71 (0.10) | 0.13 (0.03) |
| NRF | XGBoost-SI | train | 0.92 (0.01) | 0.98 (0.02) | 0.74 (0.02) | 0.13 (0.01) |
| NRF | XGBoost-SI | test | 0.88 (0.00) | 1.00 (0.01) | 0.76 (0.08) | 0.12 (0.03) |
| NRF | XGBoost-SI | val | 0.90 (0.00) | 1.00 (0.00) | 0.79 (0.07) | 0.11 (0.02) |
| NRF | XGBoost-MICE | train | 0.92 (0.00) | 0.98 (0.02) | 0.75 (0.02) | 0.13 (0.01) |
| NRF | XGBoost-MICE | test | 0.88 (0.01) | 1.00 (0.01) | 0.75 (0.08) | 0.12 (0.03) |
| NRF | XGBoost-MICE | val | 0.90 (0.00) | 1.00 (0.00) | 0.76 (0.08) | 0.12 (0.03) |
| HAEMOFIL | UAN-GVI | train | 0.91 (0.00) | 1.00 (0.00) | 0.70 (0.03) | 0.14 (0.00) |
| HAEMOFIL | UAN-GVI | test | 0.94 (0.01) | 1.00 (0.00) | 0.78 (0.07) | 0.12 (0.01) |
| HAEMOFIL | UAN-GVI | val | 0.96 (0.00) | 1.00 (0.00) | 0.80 (0.07) | 0.12 (0.01) |
| HAEMOFIL | UAN-GVI | val_p | 0.94 (0.01) | 1.00 (0.00) | 0.76 (0.13) | 0.12 (0.02) |
| HAEMOFIL | UAN-PN | train | 0.93 (0.01) | 1.00 (0.00) | 0.57 (0.04) | 0.15 (0.00) |
| HAEMOFIL | UAN-PN | test | 0.95 (0.01) | 1.00 (0.00) | 0.60 (0.15) | 0.12 (0.01) |
| HAEMOFIL | UAN-PN | val | 0.96 (0.05) | 1.00 (0.00) | 0.61 (0.15) | 0.12 (0.01) |
| HAEMOFIL | UAN-PN | val_p | 0.94 (0.04) | 1.00 (0.00) | 0.66 (0.15) | 0.13 (0.02) |
| HAEMOFIL | LR | train | 0.90 (0.00) | 1.00 (0.00) | 0.46 (0.00) | 0.14 (0.00) |
| HAEMOFIL | LR | test | 0.90 (0.01) | 1.00 (0.00) | 0.75 (0.01) | 0.13 (0.00) |
| HAEMOFIL | LR | val_p | 0.92 (0.00) | 1.00 (0.00) | 0.79 (0.01) | 0.14 (0.00) |
| HAEMOFIL | LR-SI | train | 0.92 (0.00) | 1.00 (0.00) | 0.49 (0.00) | 0.12 (0.00) |
| HAEMOFIL | LR-SI | test | 0.92 (0.00) | 1.00 (0.00) | 0.78 (0.01) | 0.11 (0.00) |
| HAEMOFIL | LR-SI | val | 0.94 (0.00) | 1.00 (0.00) | 0.81 (0.01) | 0.10 (0.00) |
| HAEMOFIL | LR-MICE | train | 0.91 (0.00) | 1.00 (0.00) | 0.50 (0.00) | 0.13 (0.00) |
| HAEMOFIL | LR-MICE | test | 0.91 (0.01) | 1.00 (0.00) | 0.78 (0.01) | 0.12 (0.00) |
| HAEMOFIL | LR-MICE | val | 0.92 (0.00) | 1.00 (0.00) | 0.79 (0.02) | 0.12 (0.00) |
| HAEMOFIL | XGBoost | train | 0.96 (0.01) | 0.99 (0.02) | 0.83 (0.03) | 0.09 (0.01) |
| HAEMOFIL | XGBoost | test | 0.93 (0.01) | 1.00 (0.00) | 0.80 (0.07) | 0.09 (0.02) |
| HAEMOFIL | XGBoost | val | 0.95 (0.01) | 1.00 (0.00) | 0.81 (0.07) | 0.09 (0.02) |
| HAEMOFIL | XGBoost-SI | train | 0.97 (0.00) | 0.99 (0.01) | 0.86 (0.01) | 0.08 (0.00) |
| HAEMOFIL | XGBoost-SI | test | 0.94 (0.01) | 1.00 (0.00) | 0.85 (0.06) | 0.08 (0.02) |
| HAEMOFIL | XGBoost-SI | val | 0.96 (0.00) | 1.00 (0.00) | 0.86 (0.05) | 0.07 (0.02) |
| HAEMOFIL | XGBoost-MICE | train | 0.97 (0.00) | 0.99 (0.01) | 0.87 (0.01) | 0.08 (0.00) |
| HAEMOFIL | XGBoost-MICE | test | 0.94 (0.01) | 1.00 (0.00) | 0.85 (0.07) | 0.08 (0.02) |
| HAEMOFIL | XGBoost-MICE | val | 0.96 (0.00) | 1.00 (0.00) | 0.83 (0.07) | 0.08 (0.02) |
| NARRT | UAN-GVI | train | 0.57 (0.09) | 1.00 (0.00) | 0.54 (0.01) | 0.25 (0.00) |
| NARRT | UAN-GVI | test | 0.56 (0.07) | 1.00 (0.00) | 0.55 (0.07) | 0.24 (0.00) |
| NARRT | UAN-GVI | val | 0.54 (0.08) | 1.00 (0.00) | 0.54 (0.07) | 0.24 (0.01) |
| NARRT | UAN-GVI | val_p | 0.56 (0.09) | 1.00 (0.00) | 0.55 (0.08) | 0.24 (0.01) |
| NARRT | UAN-PN | train | 0.53 (0.25) | 1.00 (0.00) | 0.55 (0.01) | 0.24 (0.00) |
| NARRT | UAN-PN | test | 0.53 (0.09) | 1.00 (0.00) | 0.57 (0.05) | 0.24 (0.01) |
| NARRT | UAN-PN | val | 0.40 (0.23) | 1.00 (0.00) | 0.57 (0.05) | 0.24 (0.01) |
| NARRT | UAN-PN | val_p | 0.55 (0.12) | 1.00 (0.00) | 0.57 (0.06) | 0.24 (0.01) |
| NARRT | LR | train | 0.66 (0.00) | 1.00 (0.00) | 0.49 (0.00) | 0.24 (0.00) |
| NARRT | LR | test | 0.66 (0.00) | 1.00 (0.00) | 0.49 (0.01) | 0.24 (0.00) |
| NARRT | LR | val_p | 0.65 (0.00) | 1.00 (0.00) | 0.50 (0.01) | 0.24 (0.00) |
| NARRT | LR-SI | train | 0.68 (0.00) | 1.00 (0.00) | 0.49 (0.00) | 0.23 (0.00) |
| NARRT | LR-SI | test | 0.68 (0.00) | 1.00 (0.00) | 0.49 (0.01) | 0.23 (0.00) |
| NARRT | LR-SI | val | 0.69 (0.00) | 1.00 (0.00) | 0.49 (0.01) | 0.23 (0.00) |
| NARRT | LR-MICE | train | 0.68 (0.00) | 1.00 (0.00) | 0.50 (0.00) | 0.23 (0.00) |
| NARRT | LR-MICE | test | 0.68 (0.00) | 1.00 (0.00) | 0.50 (0.01) | 0.23 (0.00) |
| NARRT | LR-MICE | val | 0.68 (0.00) | 1.00 (0.00) | 0.51 (0.00) | 0.23 (0.00) |
| NARRT | XGBoost | train | 0.72 (0.02) | 0.95 (0.07) | 0.50 (0.01) | 0.22 (0.00) |
| NARRT | XGBoost | test | 0.69 (0.01) | 0.96 (0.06) | 0.50 (0.02) | 0.22 (0.01) |
| NARRT | XGBoost | val | 0.70 (0.01) | 0.97 (0.04) | 0.48 (0.02) | 0.22 (0.01) |
| NARRT | XGBoost-SI | train | 0.75 (0.01) | 0.96 (0.06) | 0.52 (0.01) | 0.22 (0.00) |
| NARRT | XGBoost-SI | test | 0.71 (0.00) | 0.96 (0.06) | 0.50 (0.03) | 0.22 (0.01) |
| NARRT | XGBoost-SI | val | 0.72 (0.00) | 0.96 (0.06) | 0.50 (0.03) | 0.21 (0.01) |
| NARRT | XGBoost-MICE | train | 0.74 (0.01) | 0.96 (0.06) | 0.52 (0.01) | 0.22 (0.00) |
| NARRT | XGBoost-MICE | test | 0.71 (0.00) | 0.96 (0.06) | 0.50 (0.03) | 0.22 (0.01) |
| NARRT | XGBoost-MICE | val | 0.71 (0.00) | 0.96 (0.06) | 0.49 (0.02) | 0.21 (0.01) |
| INFDS | UAN-GVI | train | 0.76 (0.02) | 1.00 (0.00) | 0.59 (0.01) | 0.22 (0.00) |
| INFDS | UAN-GVI | test | 0.77 (0.04) | 1.00 (0.00) | 0.58 (0.13) | 0.21 (0.01) |
| INFDS | UAN-GVI | val | 0.84 (0.02) | 1.00 (0.00) | 0.58 (0.14) | 0.21 (0.02) |
| INFDS | UAN-GVI | val_p | 0.78 (0.02) | 1.00 (0.00) | 0.59 (0.15) | 0.21 (0.02) |
| INFDS | UAN-PN | train | 0.72 (0.12) | 1.00 (0.00) | 0.59 (0.02) | 0.22 (0.00) |
| INFDS | UAN-PN | test | 0.72 (0.15) | 1.00 (0.00) | 0.64 (0.12) | 0.21 (0.01) |
| INFDS | UAN-PN | val | 0.72 (0.18) | 1.00 (0.00) | 0.63 (0.12) | 0.22 (0.02) |
| INFDS | UAN-PN | val_p | 0.66 (0.14) | 1.00 (0.00) | 0.66 (0.13) | 0.21 (0.02) |
| INFDS | LR | train | 0.75 (0.00) | 1.00 (0.00) | 0.45 (0.00) | 0.21 (0.00) |
| INFDS | LR | test | 0.74 (0.01) | 1.00 (0.00) | 0.62 (0.04) | 0.20 (0.00) |
| INFDS | LR | val_p | 0.74 (0.00) | 1.00 (0.00) | 0.59 (0.02) | 0.21 (0.00) |
| INFDS | LR-SI | train | 0.78 (0.01) | 1.00 (0.00) | 0.47 (0.01) | 0.20 (0.00) |
| INFDS | LR-SI | test | 0.77 (0.03) | 1.00 (0.00) | 0.69 (0.02) | 0.20 (0.00) |
| INFDS | LR-SI | val | 0.80 (0.01) | 1.00 (0.00) | 0.67 (0.01) | 0.20 (0.00) |
| INFDS | LR-MICE | train | 0.78 (0.01) | 1.00 (0.00) | 0.48 (0.01) | 0.20 (0.00) |
| INFDS | LR-MICE | test | 0.76 (0.03) | 1.00 (0.00) | 0.69 (0.03) | 0.20 (0.00) |
| INFDS | LR-MICE | val | 0.77 (0.01) | 1.00 (0.00) | 0.67 (0.02) | 0.20 (0.00) |
| INFDS | XGBoost | train | 0.92 (0.02) | 0.96 (0.05) | 0.67 (0.05) | 0.15 (0.01) |
| INFDS | XGBoost | test | 0.77 (0.03) | 1.00 (0.00) | 0.71 (0.09) | 0.13 (0.04) |
| INFDS | XGBoost | val | 0.79 (0.04) | 1.00 (0.00) | 0.70 (0.10) | 0.13 (0.04) |
| INFDS | XGBoost-SI | train | 0.94 (0.01) | 0.96 (0.05) | 0.72 (0.04) | 0.14 (0.01) |
| INFDS | XGBoost-SI | test | 0.80 (0.02) | 1.00 (0.00) | 0.78 (0.09) | 0.12 (0.04) |
| INFDS | XGBoost-SI | val | 0.82 (0.03) | 1.00 (0.00) | 0.75 (0.10) | 0.13 (0.04) |
| INFDS | XGBoost-MICE | train | 0.95 (0.01) | 0.96 (0.05) | 0.72 (0.03) | 0.14 (0.01) |
| INFDS | XGBoost-MICE | test | 0.79 (0.03) | 1.00 (0.00) | 0.76 (0.09) | 0.12 (0.04) |
| INFDS | XGBoost-MICE | val | 0.82 (0.02) | 1.00 (0.00) | 0.76 (0.09) | 0.12 (0.04) |
| MORT30 | UAN-GVI | train | 0.88 (0.01) | 1.00 (0.00) | 0.68 (0.02) | 0.17 (0.00) |
| MORT30 | UAN-GVI | test | 0.91 (0.01) | 1.00 (0.00) | 0.74 (0.07) | 0.13 (0.01) |
| MORT30 | UAN-GVI | val | 0.93 (0.01) | 1.00 (0.00) | 0.77 (0.07) | 0.13 (0.01) |
| MORT30 | UAN-GVI | ev | 0.81 (0.01) | 1.00 (0.00) | 0.63 (0.10) | 0.18 (0.03) |
| MORT30 | UAN-PN | train | 0.90 (0.04) | 1.00 (0.00) | 0.58 (0.03) | 0.16 (0.00) |
| MORT30 | UAN-PN | test | 0.92 (0.06) | 1.00 (0.00) | 0.67 (0.11) | 0.14 (0.02) |
| MORT30 | UAN-PN | val | 0.90 (0.09) | 1.00 (0.00) | 0.66 (0.12) | 0.13 (0.02) |
| MORT30 | UAN-PN | ev | 0.75 (0.13) | 1.00 (0.00) | 0.67 (0.12) | 0.20 (0.03) |
| MORT30 | LR | train | 0.82 (0.00) | 0.99 (0.01) | 0.48 (0.00) | 0.18 (0.00) |
| MORT30 | LR | test | 0.82 (0.01) | 1.00 (0.00) | 0.70 (0.02) | 0.18 (0.00) |
| MORT30 | LR | val_p | 0.84 (0.00) | 1.00 (0.00) | 0.72 (0.01) | 0.18 (0.00) |
| MORT30 | LR | ev | 0.77 (0.01) | 1.00 (0.00) | 0.70 (0.01) | 0.23 (0.00) |
| MORT30 | LR-SI | train | 0.89 (0.00) | 1.00 (0.00) | 0.46 (0.00) | 0.14 (0.00) |
| MORT30 | LR-SI | test | 0.89 (0.00) | 1.00 (0.00) | 0.76 (0.02) | 0.13 (0.00) |
| MORT30 | LR-SI | val | 0.90 (0.00) | 1.00 (0.00) | 0.79 (0.02) | 0.13 (0.00) |
| MORT30 | LR-SI | ev | 0.72 (0.01) | 0.98 (0.01) | 0.63 (0.02) | 0.11 (0.00) |
| MORT30 | LR-MICE | train | 0.89 (0.00) | 1.00 (0.00) | 0.47 (0.00) | 0.15 (0.00) |
| MORT30 | LR-MICE | test | 0.89 (0.00) | 1.00 (0.00) | 0.77 (0.01) | 0.14 (0.00) |
| MORT30 | LR-MICE | val | 0.89 (0.00) | 1.00 (0.00) | 0.79 (0.02) | 0.14 (0.00) |
| MORT30 | LR-MICE | ev | 0.75 (0.00) | 0.99 (0.00) | 0.71 (0.02) | 0.17 (0.01) |
| MORT30 | XGBoost | train | 0.93 (0.03) | 0.98 (0.02) | 0.79 (0.05) | 0.12 (0.02) |
| MORT30 | XGBoost | test | 0.90 (0.03) | 1.00 (0.00) | 0.85 (0.06) | 0.10 (0.03) |
| MORT30 | XGBoost | val | 0.91 (0.03) | 1.00 (0.00) | 0.85 (0.08) | 0.09 (0.03) |
| MORT30 | XGBoost | ev | 0.80 (0.06) | 1.00 (0.00) | 0.75 (0.11) | 0.16 (0.04) |
| MORT30 | XGBoost-SI | train | 0.98 (0.00) | 0.99 (0.01) | 0.86 (0.01) | 0.08 (0.01) |
| MORT30 | XGBoost-SI | test | 0.94 (0.00) | 1.00 (0.00) | 0.90 (0.04) | 0.08 (0.02) |
| MORT30 | XGBoost-SI | val | 0.95 (0.00) | 1.00 (0.00) | 0.92 (0.04) | 0.06 (0.02) |
| MORT30 | XGBoost-SI | ev | 0.55 (0.08) | 0.99 (0.01) | 0.68 (0.12) | 0.20 (0.06) |
| MORT30 | XGBoost-MICE | train | 0.97 (0.00) | 0.99 (0.01) | 0.87 (0.01) | 0.09 (0.01) |
| MORT30 | XGBoost-MICE | test | 0.94 (0.00) | 1.00 (0.00) | 0.88 (0.05) | 0.08 (0.02) |
| MORT30 | XGBoost-MICE | val | 0.95 (0.00) | 1.00 (0.00) | 0.90 (0.04) | 0.07 (0.02) |
| MORT30 | XGBoost-MICE | ev | 0.79 (0.01) | 1.00 (0.00) | 0.84 (0.08) | 0.17 (0.04) |
