# Supplementary material for: Paying attention to cardiac surgical risk: An interpretable machine learning approach using an uncertainty-aware attentive neural network
Source: PLoS One. 2023 Aug 30;18(8):e0289930. doi: 10.1371/journal.pone.0289930 (PMC10468047; doi:10.1371/journal.pone.0289930)
Supplement: S6 Table — Statistical testing for performance differences across cross-validation. (DOCX) [file pone.0289930.s006.docx]

**S6 Table: Pairwise T-test p-values for AUC**

|  | **UAN-GVI** | **UAN-PN** | **LR** | **LR-SI** | **LR-MICE** | **XGBoost** | **XGBoost-SI** |
| --- | --- | --- | --- | --- | --- | --- | --- |
| **UAN-GVI** | 1.0 |  |  |  |  |  |  |
| **UAN-PN** | 0.7079996938531320 | 1.0 |  |  |  |  |  |
| **LR** | 3.10559394120035e-16 | 2.36247594499482e-16 | 1.0 |  |  |  |  |
| **LR-SI** | 4.61836613907583e-33 | 6.4645561298833e-28 | 3.91615223513602e-45 | 1.0 |  |  |  |
| **LR-MICE** | 9.49550821515508e-12 | 3.7724737263495e-11 | 1.8159020101146e-15 | 4.02111745384438e-25 | 1.0 |  |  |
| **XGBoost** | 0.7028109591750890 | 0.8951149718561150 | 1.90699704770538e-07 | 4.07615059767275e-49 | 1.85076087915011e-07 | 1.0 |  |
| **XGBoost-SI** | 2.8577947475902e-33 | 1.33665344916624e-26 | 1.27670685776044e-45 | 3.32282503984983e-29 | 4.24844030912582e-26 | 3.85043147460388e-73 | 1.0 |
| **XGBoost-MICE** | 5.98364913909959e-18 | 1.03185159870351e-13 | 0.1084066805279810 | 1.34405962840537e-66 | 2.59735443357287e-06 | 1.43596239083316e-15 | 1.08604352312067e-74 |
