# Supplementary material for: Paying attention to cardiac surgical risk: An interpretable machine learning approach using an uncertainty-aware attentive neural network
Source: PLoS One. 2023 Aug 30;18(8):e0289930. doi: 10.1371/journal.pone.0289930 (PMC10468047; doi:10.1371/journal.pone.0289930)
Supplement: S7 Table — Statistical testing for performance differences across cross-validation. (DOCX) [file pone.0289930.s007.docx]

**S7 Table: Pairwise T-test p-values for sensitivity**

|  | **UAN-GVI** | **UAN-PN** | **LR** | **LR-SI** | **LR-MICE** | **XGBoost** | **XGBoost-SI** |
| --- | --- | --- | --- | --- | --- | --- | --- |
| **UAN-GVI** | 1.0 |  |  |  |  |  |  |
| **UAN-PN** | 0.3662799162632990 | 1.0 |  |  |  |  |  |
| **LR** | 0.16219981475415800 | 0.004596836389786190 | 1.0 |  |  |  |  |
| **LR-SI** | 2.17432933143862e-37 | 1.47982216206779e-32 | 5.81646855019624e-62 | 1.0 |  |  |  |
| **LR-MICE** | 2.62164830334124e-09 | 6.02689282071133e-08 | 1.17491188113469e-22 | 1.46006628793523e-32 | 1.0 |  |  |
| **XGBoost** | 0.003806063786470060 | 0.010449420066986900 | 5.89651504472694e-05 | 8.89183615090674e-07 | 0.5193282902808030 | 1.0 |  |
| **XGBoost-SI** | 8.35846481644376e-07 | 1.73158884636762e-05 | 1.44336686104957e-11 | 0.00011731991571549100 | 0.02323958243423080 | 0.026953684377844300 | 1.0 |
| **XGBoost-MICE** | 0.0008230852002908760 | 0.0007215689753112120 | 2.39990996548508e-05 | 2.49817306944443e-44 | 0.001147106958603180 | 7.32753697466213e-05 | 5.66935696915812e-14 |
