# Supplementary material for: Paying attention to cardiac surgical risk: An interpretable machine learning approach using an uncertainty-aware attentive neural network
Source: PLoS One. 2023 Aug 30;18(8):e0289930. doi: 10.1371/journal.pone.0289930 (PMC10468047; doi:10.1371/journal.pone.0289930)
Supplement: S8 Table — Statistical testing for performance differences across cross-validation. (DOCX) [file pone.0289930.s008.docx]

**S8 Table: Pairwise T-test p-values for specificity**

|  | **UAN-GVI** | **UAN-PN** | **LR** | **LR-SI** | **LR-MICE** | **XGBoost** | **XGBoost-SI** |
| --- | --- | --- | --- | --- | --- | --- | --- |
| **UAN-GVI** | 1.0 |  |  |  |  |  |  |
| **UAN-PN** | 0.3466763005494350 | 1.0 |  |  |  |  |  |
| **LR** | 0.14957253442588600 | 0.424541756908109 | 1.0 |  |  |  |  |
| **LR-SI** | 2.39199506556511e-25 | 3.14956949197343e-23 | 1.42821865218192e-57 | 1.0 |  |  |  |
| **LR-MICE** | 2.68152031266308e-05 | 2.57803438791734e-05 | 1.8328790433982e-22 | 2.01440741085068e-27 | 1.0 |  |  |
| **XGBoost** | 0.00015370039542730100 | 0.00034892608328143100 | 1.43293322652749e-09 | 0.004080582498880210 | 0.1622628321522440 | 1.0 |  |
| **XGBoost-SI** | 0.8109246368633760 | 0.5784282424191230 | 0.43194113773717200 | 3.09135594595387e-08 | 0.18775373929681500 | 9.24891839213501e-06 | 1.0 |
| **XGBoost-MICE** | 0.1277948752842850 | 0.02827283140579410 | 0.00047733014437603500 | 2.31866275958172e-27 | 0.005078753313785440 | 1.83869730840141e-09 | 0.4696624113925350 |
