# Supplementary material for: Paying attention to cardiac surgical risk: An interpretable machine learning approach using an uncertainty-aware attentive neural network
Source: PLoS One. 2023 Aug 30;18(8):e0289930. doi: 10.1371/journal.pone.0289930 (PMC10468047; doi:10.1371/journal.pone.0289930)
Supplement: S9 Table — Statistical testing for performance differences across cross-validation. (DOCX) [file pone.0289930.s009.docx]

**S9 Table: Pairwise T-test p-values for AUC of high confidence samples**

|  | **UAN-GVI** | **UAN-PN** | **LR** | **LR-SI** | **LR-MICE** | **XGBoost** | **XGBoost-SI** |
| --- | --- | --- | --- | --- | --- | --- | --- |
| **UAN-GVI** | 1.0 |  |  |  |  |  |  |
| **UAN-PN** | 0.12311065962035900 | 1.0 |  |  |  |  |  |
| **LR** | 1.55303331799281e-21 | 1.76667599052208e-16 | 1.0 |  |  |  |  |
| **LR-SI** | 1.39868257091453e-38 | 6.40696856438725e-32 | 7.55028832909981e-48 | 1.0 |  |  |  |
| **LR-MICE** | 1.08629164474545e-13 | 2.01997765910825e-10 | 3.05704086042615e-09 | 1.72345000045281e-28 | 1.0 |  |  |
| **XGBoost** | 0.12549044835597300 | 0.4174049410993610 | 0.010202272641615500 | 2.26228313277374e-21 | 0.01947969405973990 | 1.0 |  |
| **XGBoost-SI** | 5.93296876158968e-13 | 3.75244801055592e-09 | 4.48813637903619e-17 | 8.10017725947809e-11 | 2.81066777226707e-08 | 3.58429681576838e-32 | 1.0 |
| **XGBoost-MICE** | 9.20280670539866e-14 | 1.32403152090229e-06 | 2.13765351475011e-21 | 7.87836488870126e-75 | 2.27464074932266e-19 | 0.6214979220663780 | 1.98473136031579e-36 |
