# Supplementary material for: Paying attention to cardiac surgical risk: An interpretable machine learning approach using an uncertainty-aware attentive neural network
Source: PLoS One. 2023 Aug 30;18(8):e0289930. doi: 10.1371/journal.pone.0289930 (PMC10468047; doi:10.1371/journal.pone.0289930)
Supplement: S10 Table — Statistical testing for performance differences across cross-validation. (DOCX) [file pone.0289930.s010.docx]

**S10 Table: Pairwise T-test p-values for AUC of aleatoric uncertainty**

|  | **UAN-GVI** | **UAN-PN** | **LR** | **LR-SI** | **LR-MICE** | **XGBoost** | **XGBoost-SI** |
| --- | --- | --- | --- | --- | --- | --- | --- |
| **UAN-GVI** | 1.0 |  |  |  |  |  |  |
| **UAN-PN** | 0.99999999 | 1.0 |  |  |  |  |  |
| **LR** | 0.0004419446036351740 | 0.004264164712500260 | 1.0 |  |  |  |  |
| **LR-SI** | 1.34275156122804e-20 | 3.29782927006525e-16 | 1.87583033259505e-31 | 1.0 |  |  |  |
| **LR-MICE** | 4.48609071890713e-08 | 5.26854367101975e-06 | 5.93473410033242e-11 | 5.98780082419822e-14 | 1.0 |  |  |
| **XGBoost** | 0.0015788729006520800 | 0.010863989621133100 | 2.30817411070646e-05 | 2.65566444857596e-07 | 0.18011933543915400 | 1.0 |  |
| **XGBoost-SI** | 0.0003591513515640850 | 0.00391138941127322 | 1.02390512623077e-06 | 0.008108533497478120 | 0.03698677882885970 | 0.051964130917597500 | 1.0 |
| **XGBoost-MICE** | 0.0016328850994162600 | 0.011118364858455500 | 0.0003697030719365240 | 1.31842608821518e-33 | 0.18730327154252600 | 2.91006145107891e-05 | 1.91131466857612e-08 |
