# Supplementary material for: Paying attention to cardiac surgical risk: An interpretable machine learning approach using an uncertainty-aware attentive neural network
Source: PLoS One. 2023 Aug 30;18(8):e0289930. doi: 10.1371/journal.pone.0289930 (PMC10468047; doi:10.1371/journal.pone.0289930)
Supplement: S11 Table — Statistical testing for performance differences across cross-validation. (DOCX) [file pone.0289930.s011.docx]

**S11 Table: Pairwise T-test p-values for AUC of epistemic uncertainty**

|  | **UAN-GVI** | **UAN-PN** | **LR** | **LR-SI** | **LR-MICE** | **XGBoost** | **XGBoost-SI** |
| --- | --- | --- | --- | --- | --- | --- | --- |
| **UAN-GVI** | 1.0 |  |  |  |  |  |  |
| **UAN-PN** | 0.18661899500759900 | 1.0 |  |  |  |  |  |
| **LR** | 0.006602441387296900 | 0.8951496048453520 | 1.0 |  |  |  |  |
| **LR-SI** | 0.8045141397651540 | 6.47782446533276e-06 | 1.80216008177673e-35 | 1.0 |  |  |  |
| **LR-MICE** | 0.06561211468846420 | 0.8602857715290960 | 0.031217597790098300 | 3.21515653860429e-23 | 1.0 |  |  |
| **XGBoost** | 0.0001565121560560130 | 0.09206970262270600 | 0.0009863498986314350 | 2.02751852551892e-11 | 0.05300894715544450 | 1.0 |  |
| **XGBoost-SI** | 0.9434994606165350 | 0.05517212429772880 | 0.0003698963835499760 | 0.6519640476209110 | 0.013142114219630500 | 2.47677780026279e-12 | 1.0 |
| **XGBoost-MICE** | 1.50502653134204e-16 | 2.68526416376944e-11 | 1.65177615033004e-31 | 1.46467063281909e-52 | 3.54831236367994e-17 | 8.1126500953802e-05 | 6.25741487561712e-30 |
