# Supplementary material for: Paying attention to cardiac surgical risk: An interpretable machine learning approach using an uncertainty-aware attentive neural network
Source: PLoS One. 2023 Aug 30;18(8):e0289930. doi: 10.1371/journal.pone.0289930 (PMC10468047; doi:10.1371/journal.pone.0289930)
Supplement: S12 Table — Statistical testing for performance differences across cross-validation. (DOCX) [file pone.0289930.s012.docx]

**S12 Table: Pairwise T-test p-values for the Brier score**

|  | **UAN-GVI** | **UAN-PN** | **LR** | **LR-SI** | **LR-MICE** | **XGBoost** | **XGBoost-SI** |
| --- | --- | --- | --- | --- | --- | --- | --- |
| **UAN-GVI** | 1.0 |  |  |  |  |  |  |
| **UAN-PN** | 0.20512796597591700 | 1.0 |  |  |  |  |  |
| **LR** | 0.0007040088945206730 | 0.5421516010706180 | 1.0 |  |  |  |  |
| **LR-SI** | 2.87824707908075e-23 | 3.37794048882195e-24 | 1.81696655605418e-59 | 1.0 |  |  |  |
| **LR-MICE** | 0.00011274173397949000 | 8.57292969676017e-06 | 3.28562055407568e-23 | 1.89029998274998e-28 | 1.0 |  |  |
| **XGBoost** | 9.75812204406086e-05 | 0.00021466837969572200 | 2.33012995475078e-11 | 0.7634508823808630 | 0.030948135181209200 | 1.0 |  |
| **XGBoost-SI** | 0.6788328270748830 | 0.3446508269005360 | 0.042133296348475400 | 3.58876553390153e-09 | 0.16469160858739900 | 5.30974566138069e-09 | 1.0 |
| **XGBoost-MICE** | 0.003010483014463650 | 0.0006096593825309060 | 9.35794567133417e-11 | 1.7064446761384e-11 | 0.7781283569997010 | 1.20838771364792e-06 | 0.007726591825054670 |
