# Supplementary material for: Paying attention to cardiac surgical risk: An interpretable machine learning approach using an uncertainty-aware attentive neural network
Source: PLoS One. 2023 Aug 30;18(8):e0289930. doi: 10.1371/journal.pone.0289930 (PMC10468047; doi:10.1371/journal.pone.0289930)

**S1 Appendix: Uncertainty-aware attention network implementation details**

The uncertainty aware attention network consists of 3 component neural networks. Firstly, there is a neural network mapping, f(x), for each input variable to a higher dimensional embedding space. Secondly, these feature embeddings are passed through an attention network, t(e), that is similar in design to a Transformer. The attention network outputs a vector in a latent space, Z. Finally, a third network, g(z), maps from the latent space to output space with uncertainty quantification built in. This network could either be a posterior network (PN) or output parameters to a beta-distribution for a generalized variational inference (GVI) approach. Algorithm 1, below,

The loss function for the posterior network is the uncertain cross-entropy (UCE) loss, which is a variant of cross-entropy loss, regularized by the entropy of the learned distribution of the true categorical distrubution. The GVI loss function is the evidence lower bound, which consists of a cross-entropy loss term and a regularization term defined as the kullback-leibler divergence.

The UAN was trained for 100 epochs with early-stopping.


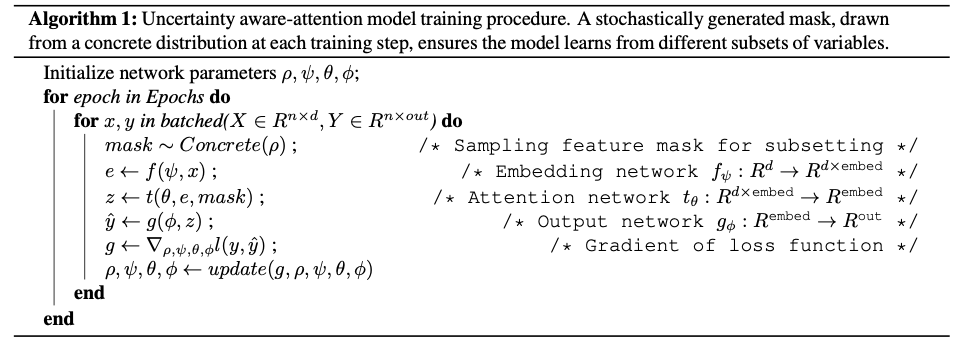

Supplement: S1 Appendix — A detailed description of the uncertainty aware network implementation. (DOCX) [file pone.0289930.s013.docx]
