# Supplementary material for: Paying attention to cardiac surgical risk: An interpretable machine learning approach using an uncertainty-aware attentive neural network
Source: PLoS One. 2023 Aug 30;18(8):e0289930. doi: 10.1371/journal.pone.0289930 (PMC10468047; doi:10.1371/journal.pone.0289930)
Supplement: S1 File — (PDF) [file pone.0289930.s014.pdf]

ANZSCTS Cardiac Surgery Database

Participant Information Sheet

Principal investigator: Professor Chris Reid
Project number: 336/20

Introduction

We would like to include you in the Australian and New Zealand Society of Cardiac and Thoracic Surgeons (ANZSCTS) Cardiac Surgery Database because you are about to have, or have recently had, heart surgery. Participating in the Database does not have any effect on the treatment you receive. The Database only collects information about your treatment and its results. Participation in this Database is voluntary. If you do not wish to take part, you do not have to. If you decide to take part and change your mind later, you are free to withdraw from the Database at any time.

The ANZSCTS Database is a clinical registry that was started in 2001. Clinical registries collect and store data from the eligible population to monitor and report on the quality of care provided to patients. The information in the ANZSCTS Database is used to assess and make sure that care for heart surgery patients in Australia and New Zealand is safe and has good outcomes. By understanding which heart procedures and methods have the most successful outcomes and what factors contribute to patient complications, we plan to improve the standard of care over time for all patients.

The Database aims to record information on every adult having heart surgery in Australia and New Zealand. Having every patient participate in the Database makes the information more useful for improving health services.

Ethics approval for the ANZSCTS Database

All research in Australia involving humans is reviewed by an independent group of people called a Human Research Ethics Committee. The ethical aspects of this research project have been approved by the Alfred Hospital Ethics Committee and Monash University Ethics Committee. This study will be carried out according to the NHMRC *National Statement on Ethical Conduct in Human Research 2007 (updated 2018)*. This statement was developed to protect the interests of people who agree to participate in human research studies.

What information is needed?

The information we collect includes your full name, date of birth, Medicare number, hospital identification number, the name of the hospital, the reason you are having heart surgery, and other information related to your health before, during and after your operation. All of this information is included in your medical records at the hospital. The hospital will monitor your progress for 30 days from the day of your surgery. Your health status at 30 days will be included in the Database. Identifying information is needed to link back to your medical records for collecting follow up information and for matching to other health related databases.

How is the information collected?

You will not be contacted by the Database staff, and are not required to do anything. The data will be collected on forms by hospital staff and entered into the Database. Information about you and your surgery will be taken from your medical records. Information about your health 30 days after surgery will be collected from your medical records or by the hospital calling you to ask a few simple questions about how you have been since your surgery.

How is the information stored and kept confidential?

The data is stored securely at Monash University in Australia using systems that meet all applicable data protection and privacy obligations. The Database security measures conform to national standards to prevent unauthorised access. All information collected for the Database that can identify you will be treated as strictly confidential. Identifying information is protected by State and Commonwealth privacy laws and would only be shared with your

permission, or in compliance with the law. Data access is limited to approved Database staff. Any future access to Database data by other organisations or researchers must be approved by a Human Research Ethics Committee and will be bound by the same privacy laws. To allow us to track long-term outcomes and changes to heart surgery over time, the information will be kept indefinitely.

How will the information be used?

We will produce reports on heart surgery outcomes for the public, government, clinical and research audiences. We expect these reports will help people understand common trends and needs that may exist for providing heart surgery services. **You will never be identified in any reports or publications from the Database.**

Researchers may use non-identified group Database data for future research projects. Non-identified means data that does not include your name, contact information, or other information that could identify you. Please be aware that by allowing your information to be stored in the Database, the non-identified information may be used for further research and quality assurance activities about the standard of care provided to patients having heart surgery. Any further research using Database data will require approval by a Human Research Ethics Committee.

The Database links the data to other hospital and government databases including the Australian Institute of Health and Welfare's National Death Index to determine long-term outcomes. All linkage activities are bound by privacy laws and must meet specific privacy and security conditions before ethics approval is granted.

Potential risks and benefits of participating

Having your data entered into the Database will not affect or alter the care and treatment you receive in any way. However, the information will allow us to continue assessing and help improve the standard of heart surgery care for all patients over time. Hospital staff involved in the data collection will have access to your medical records. The hospital and the Database will treat your information confidentially and store it securely.

You can choose not to be in the Database or ask for more information

Participation in this Database is voluntary. We understand that not everyone is comfortable having their personal information included in a Database. Your decision whether or not to take part will not affect the care you are provided in any way. If you decide to allow your information to be included in the Database but change your mind later, you are free to withdraw from participation at any stage. Please be aware that your information will be included in the Database unless you contact the Database to say that you do not want your information to be stored.

If you do not wish to have your information included in the Database, or have any questions about the Database, please contact the ANZSCTS Database Project Coordinator on the free call number: 1800 285 382

Once you have asked for your information to be removed from the Database, all your identifying information (such as name, date of birth and contact information) will be removed. However, your heart surgery information will remain in the Database anonymously.

Questions, concerns or complaints

If you have any questions about the Database, you may call the Database Project Coordinator on 1800 285 382.

If you have complaints about any aspect of the project, the way it is being conducted or any questions about being a research participant in general, then you may contact:

Complaints Officer, Office of Ethics & Research Governance, Alfred Health
Ph: (03) 9076 3619; Email: research@alfred.org.au and please quote the following project ID: 336/20
