# Supplementary material for: Paying attention to cardiac surgical risk: An interpretable machine learning approach using an uncertainty-aware attentive neural network
Source: PLoS One. 2023 Aug 30;18(8):e0289930. doi: 10.1371/journal.pone.0289930 (PMC10468047; doi:10.1371/journal.pone.0289930)
Supplement: S2 File — (PDF) [file pone.0289930.s015.pdf]

The Australian and New Zealand Society of Cardiac and Thoracic Surgeons (ANZSCTS) Database Program

Data Access Policy

Version 1.0

November 2020

Contents

Document Version Control 2
1. Preface 3
2. ANZSCTS Database Information 3
 2.1. Purpose of the ANZSCTS Database 3
 2.2. Overview of Data Collection and Management 3
 2.3. ANZSCTS Database Research Program 4
3. Confidentiality and Security of ANZSCTS Database Data 4
4. Access to Collated data from the ANZSCTS Database Program 6
 4.1. Access Statements 6
 4.2. ANZSCTS Database Program Specific Data Access Guidelines 8
5. Process for Research Requests for Access to Collated Data 10
 5.1. Application process 10
 5.2. Research Committee Review 11
6. Process for Applications to Access Hospital Level Data 13
7. Data Access Options 13
8. Fees Associated with Data Requests 13
 9.1. Provision of Data Extracts and/or Analyses 13
 9.2. Remote Data Access 13
9. Acknowledgement for Publications 14
10. Contact Information 14

Document Version Control

Version	Date	Comments/Approvals
**1.0**	16th November 2020	Initial Version<br>Approved by: ANZSCTS Database Research Committee

1. Preface

The following policy defines how collated data and/or data analyses can be accessed from the Australian and New Zealand Society of Cardiac and Thoracic Surgeons (ANZSCTS) Cardiac Surgery Database for both research and non-research purposes. This policy includes the procedures for data requests and the criteria for provision of collated data or an analysis of collated data. This policy also outlines the methods of data access and cases in which fees for such access might be applicable.

The collated ANZSCTS Database dataset is managed in line with ethics approvals, state and commonwealth privacy legislation, stringent data management procedures, and participating hospital agreements to protect against potential breaches of privacy as well as to ensure appropriate ethical integrity. The Database activities are approved by Alfred Hospital and Monash University Human Research Ethics Committees (HRECs).

2. ANZSCTS Database Information

2.1. Purpose of the ANZSCTS Database

The ANZSCTS Cardiac Surgery Database was established in 2001 and is a clinical quality registry (CQR) that collects data on the demographics, treatment, and outcomes of patients undergoing cardiothoracic surgery at contributing sites in Australia and New Zealand. The overarching aim of the Database is to maintain the provision of a high standard of care for cardiothoracic surgery patients. Accordingly, the primary function of the Database is a quality assurance program consisting of quarterly peer review of key performance indicators and feedback to each participating hospital. The secondary purpose of the Database is for use as a research dataset for projects furthering knowledge in the cardiothoracic surgery domain.

2.2. Overview of Data Collection and Management

The ANZSCTS Database in conjunction with Monash University and funding from the Victorian Department of Health and Human Services (VIC), the Centre for Excellence (NSW), Queensland Health (QLD) and individual participating hospitals, has developed and maintained a secure, web-based data collection system and data storage mechanism for analysis and reporting.

The number of sites participating in the Database Program has increased over time from six Victorian units in 2001 to 56 sites in Australia and New Zealand in 2020. Consequently, not all sites included in the current dataset have the same amount of historic data.

The registry uses an opt-off model of participation, whereby participating hospitals provide eligible patients with an information sheet about the registry and how to contact the Data Management Centre in the CCRET if they would like to have their identifying information removed from the Database.

Participating hospitals submit data on individual procedures performed at their institutions, including data about patient demographics, risk factors, operative details, and post-operative outcomes. This data is collected using predetermined procedures and standardised definitions, as outlined in the *ANZSCTS Database Data Definitions Manual*, and includes patients' identifying information. Data is collected during the hospital admission and at 30 days after the procedure.

Data is stored securely within Monash University servers and retained indefinitely. The project conforms to national operating principles for CQRs as set out by the Australian Commission on Safety and Quality in Health Care (ACSQHC). As such, the governance of the registry is in keeping with these principles. All project matters are overseen by the ANZSCTS Database Steering Committee (SC) and research projects are evaluated and monitored by the ANZSCTS Database RC. Monash University's Centre of Cardiovascular Research and Education in Therapeutics (CCRET) acts as the coordinating Data Management Centre, answering to the SC.

All hospital data remains the property of that institution. The collective registry data and data management systems operate under the custodianship of Professor Chris Reid and Monash University.

2.3. ANZSCTS Database Research Program

The ANZSCTS Database Research Program is directed by Professor Christopher Reid. The Research Program focuses on three broad themes of research pertaining to (a) risk prediction; (b) intra-operative procedural factors; and (c) improvement of clinical outcomes following cardiac surgery. To facilitate this work, the ANZSCTS Database has established links to a number of datasets including the Australian Institute of Health and Welfare's National Death Index.

The ANZSCTS Database Research Program activities are governed by the Research Committee (RC), chaired by Professor Julian Smith. RC members include (minimum of) the Chair, the Research Program Director, two nominees from the ANZSCTS Steering Committee and a nominee from the Centre of Cardiovascular Research and Education in Therapeutics (CCRET). The Committee meets quarterly to review applications and other data access or research related matters.

3. Confidentiality and Security of ANZSCTS Database Data

Data held by the ANZSCTS Database is confidential and subject to all state and commonwealth privacy laws. The full dataset contains identifiers for patients, and coded identifiers for surgeons and hospitals. Data extracts accessible by investigators will have these identifying variables removed, except in rare circumstances (assessed on a case-by-case basis by the RC).

Data access and use must be in keeping with the information provided to registry participants when they are given the opportunity to opt-out of inclusion in the Database, as outlined below:

> *'The data is stored securely at Monash University in Australia using systems that meet all applicable data protection and privacy obligations. The Database security measures conform to national standards to prevent unauthorised access. All information collected for the Database that can identify you will be treated as strictly confidential. Identifying information is protected by State and Commonwealth privacy laws and would only be shared with your permission, or in compliance with the law. Data access is limited to approved Database staff. Any future access to Database data by other organisations or researchers must be approved by a Human Research Ethics Committee and will be bound by the same privacy laws. To allow us to track long-term outcomes and changes to heart surgery over time, the information will be kept indefinitely.*
>
> *We will produce reports on heart surgery outcomes for the public, government, clinical and research audiences. We expect these reports will help people understand common trends and needs that may exist for providing heart surgery services.* ***You will never be identified in any reports or publications from the Database.***
>
> *Researchers may use non-identified group Database data for future research projects. Non-identified means data that does not include your name, contact information, or other information that could identify you. Please be aware that by allowing your information to be stored in the Database, the non-identified information may be used for further research and quality assurance activities about the standard of care provided to patients having heart surgery. Any further research using Database data will require approval by a Human Research Ethics Committee.'*
>
> - Excerpt from the ANZSCTS Database Patient Information Sheet v8.0 (17th June 2020)

Accordingly, patient identifiers will never be made available directly to investigators and data cannot leave the secure Monash University environment.

Data security and storage remains the responsibility of the Data Management Centre at CCRET, Monash University. In order to maintain data security and integrity, all data analyses will be conducted and/or supervised by the ANZSCTS Database Program Manager or delegated staff at the Data Management Centre. All data will remain under the custodianship of the ANZSCTS Database Data Custodian. Approved requests for data will be provisioned by access to the data in a secure environment either on-site at CCRET (resources permitting), or through remote platforms (refer to Section 7 for more information).

In addition to providing HREC approval, investigators seeking access to data extracts will be required to sign a confidentiality statement (*Confidentiality Statement for Accessing ANZSCTS Database Data*) prior to access being granted.

4. Access to Collated data from the ANZSCTS Database Program

The ANZSCTS Database encourages collaborative ventures. The Data Custodian and RC envisage that ANZSCTS Database data will be of use to a large number of organisations, investigators and policy writers. Accordingly, sharing ANZSCTS Database data with relevant public, clinical and academic communities is an objective of the registry. Interested parties may present requests for access to a non-identifiable data extract or an analysis of data for the purposes of academic research, clinical review, planning or scientific investigation.

Access to data is subject to the approval of the ANZSCTS Database Research Committee (RC) and/or Steering Committee (in some cases standing approval exists), depending on the type of request. When considering the approval of access to ANZSCTS Database data for research, the RC seeks to balance the importance of privacy protection and the scientific merit of the proposal and public health interest from the proposed research.

A site remains the owner of the data they contribute and has access to this data via the online web system. Data Managers can export a full copy of the dataset, as can Heads of Unit at public hospitals. Only the ANZSCTS Database Program Manager and project team have direct access to the entire cohort of raw data.

Variables identifying individual surgeons, hospitals or hospital state/country are removed from data extracts. Investigators that require a coded variable to separate surgeons, hospitals, or hospital locations for the purpose of their analyses should clearly state this in the project application and justify the reasons. Combinations of coded variables that would allow reidentification of individual surgeons or hospitals will not be provided.

The Database regularly performs linkages with the Australian Institute of Health and Welfare's National Death Index to obtain long-term mortality data. Investigators seeking this information in their data extract should clearly state this in the project application.

All requests must be complete and submitted using the relevant proforma before consideration by the relevant Committee or Program Manager, and are subject to the guidelines and policies in this document. The ANZSCTS Database encourages the use of collated, non-identifiable data for appropriate research but holds the rights to reject or revoke access if the terms of this data access policy are exploited.

4.1. Access Statements

All requests for access to data or for analyses of data are subject to the following:

1. Access to the data is subject to the Specific Access Guidelines outlined in Section 4.2 of this document.
2. The use of data for research purposes must receive approval from the ANZSCTS Database RC. Research related requests will require specific approval from an NHMRC registered HREC prior

to access to the data being granted. Provisional project approval can be given by the ANZSCTS RC for successful applications, pending final HREC approval.

3. Investigators wanting to access data are required to sign a confidentiality statement (*Confidentiality Statement for Accessing ANZSCTS Database Data)* before access will be granted.

4. Any further research arising from an approved research application must obtain additional, separate RC and HREC approval.

5. The primary contact for the project will be required to submit regular progress reports.

6. To ensure that the data and any limitations in scope or quality of the ANZSCTS Database data have been properly understood by the user, pre-publication drafts or presentations of any derivative works must be submitted to the ANZSCTS Database RC for review and potential advice on data interpretation and Database representation. The ANZSCTS Database reserves the right to dissociate itself from conclusions drawn from the data, if deemed necessary.

7. Any material or manuscript to be published using ANZSCTS Database data must contain the appropriate acknowledgement of the ANZSCTS Database Program and its funding sources and contributors. Preferred wording for the acknowledgement is provided in Section 9 of this policy.

8. The ANZSCTS Database expends significant time, effort and other resources in collecting, cleaning and preparing the data for research. Because the binational dataset comes from multiple centres, the investigators connected to the ANZSCTS Database are also exclusively positioned to take responsibility for the quality and accuracy of the data. For these reasons, consistent with the ICMJE authorship criteria, it is expected that any manuscript arising from an ANZSCTS Database research data extract include a minimum of two authors from the ANZSCTS Database RC and/or CCRET.

9. Data with patient identifiers will not be made directly available to investigators. With a non-identifiable dataset, there still remains a very minor risk of reidentification of patients or hospitals. All users of data from the ANZSCTS Database must agree not to use that data, alone or in conjunction with any other information, to attempt to identify patients.

10. If an investigator requires linkage of other datasets to ANZSCTS Database data, the linkage must be performed within the CCRET or at either an Accredited Integrating Authority or a Population Health Research Network Australian Data Linkage Unit. Patient identifiers will not be released directly to investigators under any circumstances.

11. Provision of access to non-identifiable data or analyses will depend on the available resources at the time of request. It is under the discretion of the Data Custodian, ANZSCTS Database Program Manager and RC to prioritise the order of data requests, upon approval.

12. Investigators are encouraged to complete their research in a timely manner. Data analysis should be completed within 12 months of commencement of the project. If the project exceeds 12 months duration, the topic of research will no longer be reserved for exclusive use by the investigators. Presentation and publication of outcomes is expected following analysis, and manuscripts should be submitted within one year following completion of analysis.

13. Investigators affiliated with projects that have exceeded 24 months in duration will not have new projects considered unless sufficient progress in the existing projects has been demonstrated.

14. Requests for data access or release of information pertaining to individual surgeons or hospitals will not be granted without appropriate authorisations, in line with the relevant ethics approvals, hospital agreements, and the Database's Declaration as a Quality Assurance Activity (under section 124X of the Health Insurance Act 1973).

15. Data summaries and analyses provided by the CCRET may incur a fee to cover the costs for staff to undertake this work. See the 'Fees for Provision of Data' statement (Section 8 of this document) for an explanation of the fees.

16. Investigators are responsible for the expenses associated with the use of remote data access systems. These fees are outlined in Section 8 of this document.

17. RC meetings are held four times per year and data cannot be extracted until approval is given by the RC. Under exceptional circumstances, when data is required earlier, the ANZSCTS Database Program Manager may request an 'out of session' approval by the RC electronically to consider specific data requests.

18. All data requests must be formally lodged, using the appropriate proforma (as outlined in Section 4.2). Contact the ANZSCTS Database team for proforma templates via email: anzscts@monash.edu.

4.2. ANZSCTS Database Program Specific Data Access Guidelines

Category 1. Basic summary data

Where only basic summary data is requested, this information can be provided by ANZSCTS Database Program staff. Such provision of data does not require RC approval but the ANZSCTS Database will require a formal request in writing and will keep a record of such requests. Investigators are required to submit the *ANZSCTS Database Hospital and Summary Data Requests* proforma. The ANZSCTS Database SC will be provided with a summary of such requests.

Category 2. Collated non-identifiable data for research

All research project applications must be made in writing to the ANZSCTS Database Program Manager who will submit the data request to the next RC meeting. Investigators are required to submit an Expression of Interest (EOI) using the *Expression of Interest for Research using ANZSCTS Database Data* proforma to the Program Manager for review. Investigators will be invited to complete a full application using the *ANZSCTS Database Research Request for Data or Analysis of Data* proforma if the project topic does not duplicate existing research and the dataset is deemed sufficient to address the research question. A decision on whether to provide access to the data will be made by the RC following advice from the Program Manager. The requester(s) must submit the relevant ethics application(s), provide evidence of approval, and sign a confidentiality statement (*Confidentiality Statement for Accessing ANZSCTS Database Data*) prior to gaining access to the data. At no stage will data that could identify individual clinicians and/or patients be provided.

Category 3. Ad hoc analyses performed by the ANZSCTS Database

Investigators may request the ANZSCTS Database undertake specific ad hoc data analyses. In all cases, the investigators would subsequently be provided with resulting aggregate data only. The request process follows the same path as for Category 2. Investigators are required to submit an EOI using the *Expression of Interest for Research using ANZSCTS Database Data* proforma to the Program Manager for review. Investigators will be invited to complete a full application using the *ANZSCTS Database Research Request for Data or Analysis of Data* proforma if the project topic does not duplicate existing research and the dataset is deemed sufficient to address the research question. A decision on whether to undertake the analysis work will be made by the RC following advice from the Program Manager. The requester(s) must submit the relevant ethics application(s) required and provide evidence of approval prior to the release of analysis output(s).

Category 4. Linkage projects

If an investigator requires linkage of other datasets to ANZSCTS Database data, the linkage must be performed within the CCRET or at either an Accredited Integrating Authority or a Population Health Research Network Australian Data Linkage Unit. Patient identifiers will not be released directly to investigators under any circumstances. Investigators are encouraged to contact the Program Manager early in their planning and/or submit an EOI using the *Expression of Interest for Research using ANZSCTS Database Data* proforma to the Program Manager for review. Investigators will be invited to complete a full application using the *ANZSCTS Database Research Request for Data or Analysis of Data* proforma if the project is deemed feasible. A decision on whether to undertake the analysis work will be made by the RC following advice from the Program Manager. All such linkage projects will require ethics committee approval and a Memorandum of Understanding or legal agreement between the custodians of the included datasets.

Category 5. Hospital-specific performance information

If a hospital or its representative makes a specific request for its own performance information, beyond what is available in the web system, quarterly and/or annual reports, the ANZSCTS Database will provide such information. No case-level data that could specifically identify a patient will be provided. All such requests should be made in writing to the ANZSCTS Database Program Manager using the *ANZSCTS Database Hospital and Summary Data Requests* proforma, and are bound by the timelines and processes listed in this document. Whilst such information requests do not require committee approval, the Program Manager will notify the SC and RC of these requests.

5. Process for Research Requests for Access to Collated Data

5.1. Application process

Investigators are encouraged to contact the Program Manager when they are first considering a project using ANZSCTS Database data to discuss potential research questions, the application process, and the particulars of the dataset. Engaging with the Database early can prevent investigators from progressing with projects that are not feasible due to project duplication or limitations of the dataset.

The formal process for applications for research projects (categories 2 – 4 in Section 4.2) is detailed below and in Figure 1.

Initial formal requests should be made in writing to the Program Manager using the *Expression of Interest for Research using ANZSCTS Database Data* proforma (available on the ANZSCTS Database website: https://anzscts.org/database/research/).

The EOI includes the following information:

- Project title
- Principal investigator and co-investigators*
- Brief description of the project (<200 words)
- Knowledge gap addressed by the project (<200 words)
- Project timeline

**Note: the project should include at least one ANZSCTS member as an investigator. Researchers without the support of an ANZSCTS member should contact the Program Manager to discuss options.*

If the Program Manager determines the proposed project does not overlap with a current or recently completed project and the research question can be addressed using the ANZSCTS Database data, the investigators will be invited to submit a full application using the *ANZSCTS Database Research Request for Data or Analysis of Data* proforma. The full application includes more detail than the EOI, and a full research plan covering the background, specific aims and objectives, hypotheses, methods and detailed statistical analysis plan, and proposed publication/presentation plan.

The application should also address the following:

- The degree of analytical and statistical support that the investigators anticipate will be necessary from the ANZSCTS Database Program;
- The exact nature of the dataset to be developed and used in the analysis;
- The format of the dataset to be used in the analysis, including medium and software requirements;
- The resources available for the project at the investigator's institution;

- Details of the NHMRC registered HREC to which the project has been submitted to or that the investigator intends to submit to, attaching copies of letters of approvals and conditions, where appropriate;
- The timeframe for completion of the project;
- Which scientific journal(s) the completed manuscript(s) are likely to be submitted and/or information about any conferences or seminars that the Investigator(s) intend to submit the project.

The Principal Investigator must sign the application, thereby confirming they have read and understood the present Data Access Policy and agree to its terms.

The ANZSCTS Database RC will review the application at the next meeting and their decision will be reported back to the investigators generally within two weeks of the meeting. There are limits on the number of projects that can be reviewed each meeting and if the number of applications exceed these, projects will be reviewed in order of submission.

Following full or provisional approval, a meeting will be set up with the Program Manager and the key investigators to discuss data preparation and access arrangements, and the analysis plan. Once evidence of ethics approval has been provided, fees for remote data access addressed, and a confidentiality statement signed for each investigator accessing the data, access to the data extract will be provided.

CCRET and the RC work collaboratively with investigators and encourage discussion about variables and interpretation of results throughout the project. Investigators will be asked to submit regular progress reports until the project is completed. As outlined above, all manuscripts must be reviewed by the RC prior to submission, and include authors from the ANZSCTS Database.

5.2. Research Committee Review

Requests are reviewed for scientific merit and alignment to the RC's research priority areas. In particular, the RC focus on whether the project novelty and aims are reasonable and of interest to the broader community, whether the design and methodology are appropriate and achievable, the feasibility of the timeline, and whether the project team are well suited to support the research work. Consideration is also given to the level of CCRET resources required to fulfil the request and the track record of investigators that have previously used ANZSCTS Database data for research.

Figure 1. ANZSCTS Database Data Requests for Research Process

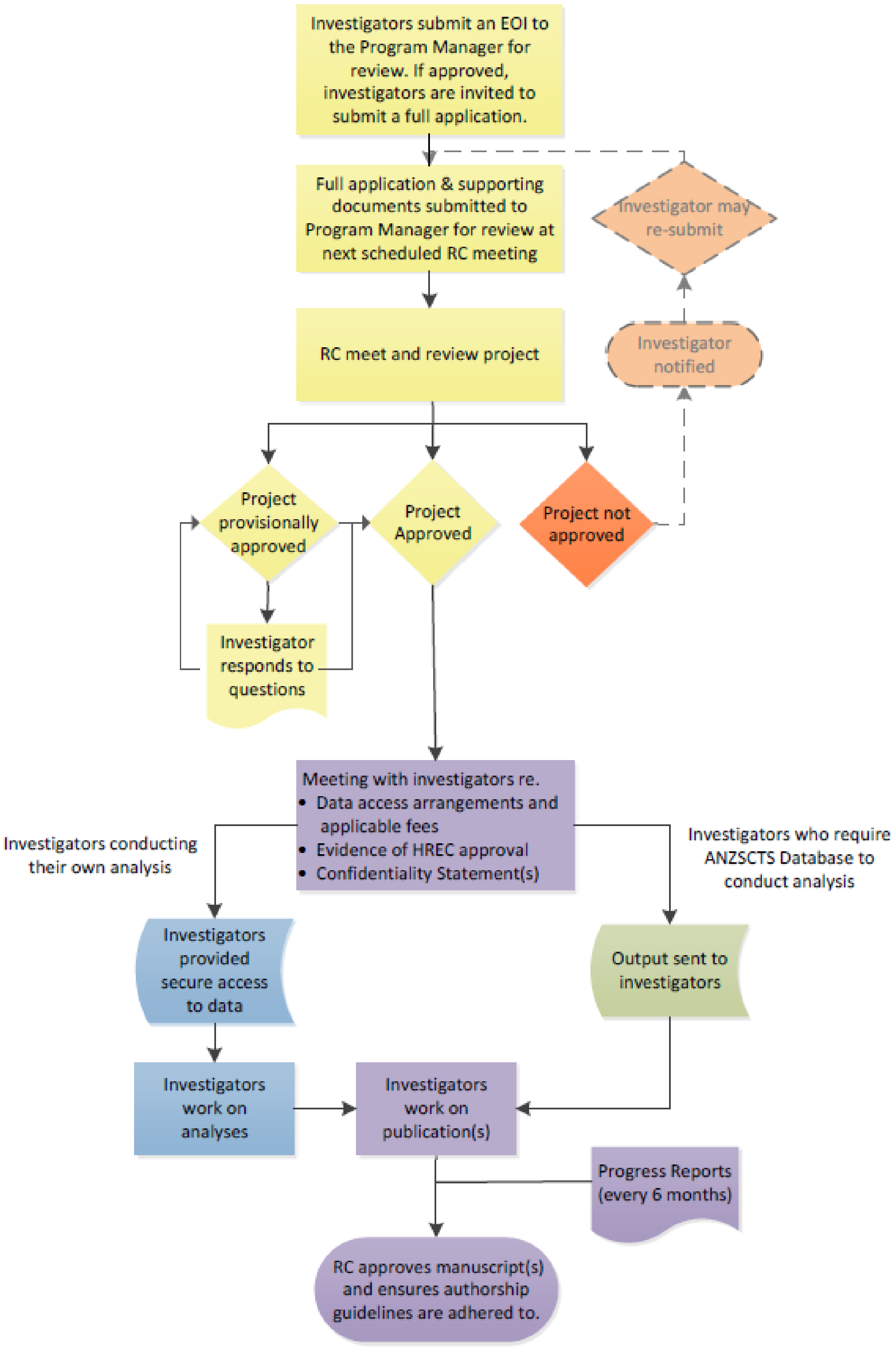

6. Process for Applications to Access Hospital Level Data

Requests for access to data or data analyses for purposes other than research (categories 1 and 5 in Section 4.2) should be submitted to the Program Manager using the *ANZSCTS Database Hospital and Summary Data Requests* proforma. These requests are facilitated by the Program Manager and do not routinely go through the RC for approval, but summaries are provided to the SC. The exact pathway of approval may vary, depending on the specific requirements of the request. There are no set review meetings or deadlines for these requests, but provision of data analyses will be bound by the timelines and processes listed in this document.

7. Data Access Options

Once research project approval has been granted, CCRET utilises a system that allows non-Monash University investigators to remotely and securely access and analyse data from ANZSCTS Database, which is known as the Monash Secure eResearch Platform (SeRP). This platform allows users to analyse data on secure virtual machines from any location, and the resulting research outputs can be exported from the server while the data file remains locked. Set-ups can be customised to meet the specific software and user configurations required for each project. The Monash SeRP is associated with set-up and maintenance fees, and these are outlined in Section 8.

A copy of the dataset will not be released to the project investigators outside of this environment under any circumstances.

8. Fees Associated with Data Requests

8.1. Provision of Data Extracts and/or Analyses

Provision of data extracts and/or analyses may incur a fee, particularly for non-collaborative work. Following review, the ANZSCTS Database RC and/or Program Manager will provide recommendations as to whether fees will be applicable and the overall cost, determined on a case-by-case basis. The overall cost will be calculated on a cost recovery basis and will be based on the time and effort involved in undertaking the work. The charge per hour of work (or part thereof) is $150 (ex GST). The minimum number of hours charged is two. This fee is for basic tabulations and data extractions only. When detailed analysis of data is required (e.g. statistical comparisons or modelling) a higher rate will be charged. The requestor will be advised in writing and will need to agree in order for the work to proceed.

8.2. Remote Data Access

If investigators require remote data access via the Monash SeRP, they will be responsible for the associated expenses. A $750 set-up fee applies per project to establish the secure research environment. This includes the set-up of an external Monash user account, use of statistical software, and preparation of the dataset. A further $500 fee applies per year that the secure

research environment is used, for licensing and storage expenses. Accordingly, the first year of a project in the Monash SeRP incurs a fee of $1250, total, and each subsequent year is $500. Fees are charged prospectively, and must be paid before data access can be provided.

9. Acknowledgement for Publications

A condition of use for the ANZSCTS Database data is that any material or manuscript to be published must contain the appropriate acknowledgement of the ANZSCTS Database Program and its funding sources and contributors. Preferred wording for the acknowledgement is:

> *The ANZSCTS Cardiac Surgery Database Program is funded by the Department of Health (VIC), the Clinical Excellence Commission (NSW), Queensland Health (QLD), and funding from individual Units. ANZSCTS Database Research activities are supported through a National Health and Medical Research Council Senior Research Fellowship and Program Grant awarded to C.M. Reid. The Database thanks all of the investigators, data managers, and institutions that participate in the Program.*

If investigators intend on using a different acknowledgement, this should be discussed with the ANZSCTS Database Program Manager and/or RC prior to submission.

10. Contact Information

If you have any questions about this Data Access Policy or the application process for accessing ANZSCTS Database data, please contact the ANZSCTS Database Team:

Email: anzscts@monash.edu

Phone: 03 9903 0518
