# Supplementary material for: Paying attention to cardiac surgical risk: An interpretable machine learning approach using an uncertainty-aware attentive neural network
Source: PLoS One. 2023 Aug 30;18(8):e0289930. doi: 10.1371/journal.pone.0289930 (PMC10468047; doi:10.1371/journal.pone.0289930)
Supplement: Attachment — Submitted filename: ReviewerComments_Response.docx [file pone.0289930.s016.docx]

**EDITORIAL COMMENTS:**

When submitting your revision, we need you to address these additional requirements.

**1. Please ensure that your manuscript meets PLOS ONE's style requirements, including those for file naming. The PLOS ONE style templates can be found at** <https://journals.plos.org/plosone/s/file?id=wjVg/PLOSOne_formatting_sample_main_body.pdf> and

<https://journals.plos.org/plosone/s/file?id=ba62/PLOSOne_formatting_sample_title_authors_affiliations.pdf>

*Response:*

Thank you for the above resources. The manuscript has been formatted accordingly.

**2. Will the web application be publicly available upon publication?** Please note that PLOS ONE has specific guidelines on code sharing for submissions in which author-generated code underpins the findings in the manuscript. In these cases, all author-generated code must be made available without restrictions upon publication of the work. Please review our guidelines at <https://journals.plos.org/plosone/s/materials-and-software-sharing#loc-sharing-code> and ensure that your code is shared in a way that follows best practice and facilitates reproducibility and reuse."

*Response:*

The code base for generating the web application and running the experiments will be available as a public GitHub repository (https://github.com/jahanpd/UAN).

**3. "Please amend your current ethics statement to address the following concerns:**

a) Did participants provide their written or verbal informed consent to participate in this study?

b) If consent was verbal, please explain i) why written consent was not obtained, ii) how you documented participant consent, and iii) whether the ethics committees/IRB approved this consent procedure.

*Response:*

The ethics statement has been amended as requested to address the question of consent. The ANZSCTS National Database program data policy and patient information sheet have been included as supplementary materials. See lines 210-213.

**4. Thank you for stating the following in the Acknowledgments Section of your manuscript:**

"The ANZSCTS Cardiac Surgery Database Program is funded by the Department of Health (Victoria), the Clinical Excellence Commission (NSW), Queensland Health (QLD), and funding from individual cardiac surgical units participating in the registry. ANZSCTS Database Research activities are supported through a National Health and Medical Research Council Principal Research Fellowship (APP 1136372) and Program Grant (APP 1092642) awarded to C.M. Reid. The Database thanks all of the investigators, data managers, and institutions that participate in the Program."

We note that you have provided funding information that is not currently declared in your Funding Statement. However, funding information should not appear in the Acknowledgments section or other areas of your manuscript. We will only publish funding information present in the Funding Statement section of the online submission form.

Please remove any funding-related text from the manuscript and let us know how you would like to update your Funding Statement. Currently, your Funding Statement reads as follows:

"The author(s) received no specific funding for this work."

Please include your amended statements within your cover letter; we will change the online submission form on your behalf.

*Response:*

Thank you for highlighting this apparent discrepancy. It should be noted that the database operates under a grant structure with its own ethics approval, whereas the research of this paper is separate from the operations of the database organization. This work received no specific funding. We have addressed this separation of concerns in our funding statement and removed the funding statement from the acknowledgments.

**REVIEWER COMMENTS (Tasks Highlighted):**

5. Review Comments to the Author

Please use the space provided to explain your answers to the questions above. You may also include additional comments for the author, including concerns about dual publication, research ethics, or publication ethics. (Please upload your review as an attachment if it exceeds 20,000 characters)

**Reviewer #1:**

*• There are many redundant sentences in the abstract, and the logic is also not clear enough. Please refine the content of the abstract again. Author need to make clear the research significance of the article, the research process, the experimental results and conclusions.*

Response:

Thank you for your feedback. The abstract has been rewritten to address these concerns and fulfill PLOS One formatting requirements.

*• The keywords need to be extracted again, and it could be best to reflect the characteristics of this article.*

Response:

Thank you for your comments. We have revised our keywords.

*• The introduction of the article is complex and tedious about the modern approaches, so it is necessary to highlight the research status at home and abroad, and simplify the background of the article.*

Response:

Thank you for your feedback. We have amended our introduction to simplify the discussion of alternative approaches and moved some of the method details to the methods section.

*• In this manuscript, the explanation of flow diagram in Figure 1 is not enough, and the author needs to give a more detailed explanation to facilitate the understanding of readers.*

Response:

Thank you for your feedback. In-text reference to Figure 1 has been made on line 140 in order to provide in-text context to the visualized data processing pipeline in Figure 1.

*• The analysis of the experimental data in Table 3 can be added to improve the uncertainty calibration calculation.*

Response:

Thank you for your feedback. We have added references to Table 3 at lines 232 and 236.

*• The conclusion of this manuscript needs to be reorganized by highlighting the research results of this research work, not too much introduction of future research methods.*

Response:

Thank you for your feedback. The discussion and conclusion have been edited and reorganized to focus more on the research presented.

*• There are grammatical errors in this article, which need to be corrected after careful checking.*

Response:

Thank you for your feedback. We have parsed our work and corrected our grammar.

*• Carefully check the references of the article and also ensure the citation is in the right place.*

Response:

Thank you for your feedback. We have re-evaluated the references list.

*• The objectives of your work need to be clearly stated in different sections.*

Response:

Thank you for your feedback. Aims and hypotheses have been altered to be more clear.

*• Author needs to highlight the novelty of this research work in clear manner.*

Response:

Thank you for your feedback. The novelty of this work has been highlighted on lines 267, 281, 341. Additionally, we hope that the further supplementary materials provided to explain the model in greater detail aid the readers in appreciating the novelty of our approach.

**Reviewer #2: The given manuscript by Penny-Dimri et al. highlights a relevant subject and presents a potentially beneficial and useful method. The results seem convincing. However, some issues need to be addressed before the manuscript can be published.**

Major issues (in decreasing order of importance):

*- The UAN approach should be described in detail, as this is not a commonly used method. Please provide details about the architecture, its internals, and the training procedure.*

Response:

Thank you for your feedback. We have added further details regarding the model design and the training algorithm in the supplemental materials (S1 Appendix) with in-text reference at line 162.

*- In Tables 2 and 3, the best results are written in bold. Does that mean that they are simply the best or does that mean that the bold results are significantly better than other results? I strongly suggest testing for significance and describing the employed statistical procedure in detail.*

Response:

Thank you for your suggestion. The bold results are simply the best performing. Regarding the application of a statistical test on cross-validation, this is a relatively controversial topic. While cross-validation and other bootstrap methods are widely used to assess the generalization performance of machine learning and statistical models, the process is well known to not produce independent samples. This arises as each fold will share data across training and test sets. As such, applying traditional statistical tests, such as a student's T-test will produce excess type 1 error. While there are corrected tests and cross-validation schemes that purport to improve the power of these tests, we suggest that these come burdened with their own assumptions and do not solve the underlying problem (some references that highlight the problems and approaches include: <https://doi.org/10.48550/arXiv.2104.00673>, <https://doi.org/10.1023/A:1024068626366>, [https://doi.org/](https://doi.org/10.1023/A:1024068626366)[10.1162/neco.1997.9.6.1245](http://dx.doi.org/10.1162/neco.1997.9.6.1245), [https://doi.org/](https://doi.org/10.1023/A:1024068626366)[10.1162/089976698300017197](https://doi.org/10.1162/089976698300017197) [https://doi.org/](https://doi.org/10.1023/A:1024068626366)10.1007/978-3-540-24775-3_3). Applying a statistical test may give an unreliable p-value and we fear reporting such results would give the reader a false assurance. We also note that it is quite common in ML literature to simply report the raw performance. We have chosen to report the mean of the cross-validation with an accompanying measure of the variance. We believe this gives readers enough information to assess the distribution of performance for each model.

*- It is imperative (otherwise a bias is introduced that renders the results invalid) that the calibration/training of missing value imputation methods is only done on the training set(s). Was that the case in the third strategy mentioned in the manuscript? It did not become entirely clear. Please provide more detail!*

Response:

Thank you for your feedback. Indeed missing value imputation was performed only on the training set data during cross-validation. This has been specified in-text at line 181, and is verifiable from the provided codebase.

*- Include more recent references to works dealing with machine learning in cardiac surgery, e.g.:*

Response:

Thank you for your suggestions. We have cited two recent meta-analyses, the latter which includes Bodenhofer et al. We have added the additional citations as requested, however our phrasing remains conservative as the most recent meta-analysis on this topic failed to find a significant advantage of ML over LR (<https://doi.org/10.1111/jocs.16842>). Lundberg and Lee and Sharme and Conte have been cited where we reference explainability on line 279.

Minor issues:

*- The figures have very poor resolutions. In particular, the heatmaps in Fig. 2 are illegible. Please fix this!*

Response:

Thank you for your feedback. We have endeavored to reformat the images as per PLOS One guidelines and using the PACE online tool to ensure adequate formatting.
