# Supplementary material for: Paying attention to cardiac surgical risk: An interpretable machine learning approach using an uncertainty-aware attentive neural network
Source: PLoS One. 2023 Aug 30;18(8):e0289930. doi: 10.1371/journal.pone.0289930 (PMC10468047; doi:10.1371/journal.pone.0289930)
Supplement: Attachment — Submitted filename: ReviewerComments_Response.docx [file pone.0289930.s017.docx]

**Additional Editor Comments:**

Please carefully address the comments from Reviewer 2 and ensure the claim of this paper is not overstated and biased, as pointed out by Reviewer 2. I also recommend the authors perform the additional test Reviewer 2 requested.

Response:

Thank-you for your feedback. Statistical testing has been performed and is available in the supplemental materials (see response to reviewer 2).

**Reviewers' comments:**

Please use the space provided to explain your answers to the questions above. You may also include additional comments for the author, including concerns about dual publication, research ethics, or publication ethics. (Please upload your review as an attachment if it exceeds 20,000 characters)

Reviewer #2: Most of my comments have properly been taken into account by the authors, so I can recommend the manuscript for publication now.

There are two issues, however, that I still want to comment on:

1) I agree that significance of cross validation results are a subtle topic. I acknowledge that p-values might be inflated, so the tests might have a higher type I error rate. It is a fact, though, that such tests are still quite common in literature. Let me make this point clear: I was not so much afraid of insignificant differences being falsely flagged as significant. My concern would rather have been the following: If a result marked in bold would fail the test for significance, then it has no justification to be reported as a serious advancement. So I was rather afraid of falsely announcing an algorithm as better than the rest although it is actually on par with another. Apart from that, I have no doubt - looking at the numbers and the standard deviations in parentheses - that the significance test would be successful. So I suggest the authors either consider performing the test (despite its weaknesses and limitations) or provide a short argumentation in the manuscript why they have not done it (even though it is quite commeon).

Thank-you for your ongoing review. We have provided statistical testing using T-tests and amended the manuscript to reflect this at lines 182 and 240. Tables of p-values for the pairwise T-tests are provided in the supplementary materials.

2) Regarding the citations on machine learning in cardiac surgery that I suggested: I am not in the position to force the authors to cite particular papers, so I accept that they have not followed my suggestion. However, since they mention their own meta-study (which has very much attracted my interest when it appeared), I want to take this opportunity to comment on this work in relation to the present manuscript under review. First of all, I pretty much disagree with their earlier meta-study. I think it deals with an ill-posed question. Nobody every claimed that ML gives a general edge over LR. I fully agree that there might be some situations in which LR is sufficient or even advisable (less overfitting, better interpretability). However, there might also be situations in which ML indeed is advantageous, in particular in situations with complex, non-linear interactions between the input features. I try not to be too polemic about this issue, but the meta-study appears to me like asking whether there is evidence that trucks are better than cars. Well, in many cases, no. But there are some case where the additional power and loading capacity of a truck is of great advantage. It depends on the situation. It is now really ironic/funny/whatever that the authors of exactly this meta-study try to get a paper about ML in cardical surgery published. No matter whether you cite your own meta-study, I think this asks for an explanation.

Response:

We thank the reviewer for their feedback. Additionally, we appreciate their analysis of our previous work and wholeheartedly agree with their conclusion. In particular, we wish to draw their attention to the discussion of the meta-analysis where we make precisely the same point. This is to say that although ML does not outperform LR as defined by the C-index, there are many reasons to consider using an ML model in non-linear settings such as peri-operative risk prediction. We suggest that ML can add value to risk prediction beyond raw performance. We make explicit the advantages of using explanatory modelling, such as with Shapley values. Furthermore, we believe that our submission under review adds to this argument by showing the flexibility to missing data, and unique explanatory visualization, which our novel ML approach provides.
